# Distinct subspecies or phenotypic plasticity? Genetic and morphological differentiation of mountain honey bees in East Africa

**DOI:** 10.1002/ece3.711

**Published:** 2013-08-06

**Authors:** Karl Gruber, Caspar Schöning, Marianne Otte, Wanja Kinuthia, Martin Hasselmann

**Affiliations:** 1 Evolutionsgenetik, Heinrich-Heine Universität DüsseldorfDüsseldorf, Germany; 2 Hohen Neuendorf, Länderinstitut für BienenkundeHohen Neuendorf, Germany; 3 National Museums of KenyaNairobi, Kenya

**Keywords:** Adaptation, East Africa, gene flow, honey bee populations, phenotypic differentiation, phenotypic plasticity

## Abstract

Identifying the forces shaping intraspecific phenotypic and genotypic divergence are of key importance in evolutionary biology. Phenotypic divergence may result from local adaptation or, especially in species with strong gene flow, from pronounced phenotypic plasticity. Here, we examine morphological and genetic divergence among populations of the western honey bee *Apis mellifera* in the topographically heterogeneous East African region. The currently accepted “mountain refugia hypothesis” states that populations living in disjunct montane forests belong to a different lineage than those in savanna habitats surrounding these forests. We obtained microsatellite data, mitochondrial sequences, and morphometric data from worker honey bees collected from feral colonies in three montane forests and corresponding neighboring savanna regions in Kenya. Honey bee colonies from montane forests showed distinct worker morphology compared with colonies in savanna areas. Mitochondrial sequence data did not support the existence of the two currently accepted subspecies. Furthermore, analyses of the microsatellite data with a Bayesian clustering method did not support the existence of two source populations as it would be expected under the mountain refugia scenario. Our findings suggest that phenotypic plasticity rather than distinct ancestry is the leading cause behind the phenotypic divergence observed between montane forest and savanna honey bees. Our study thus corroborates the idea that high gene flow may select for increased plasticity.

## Introduction

Understanding the mechanisms and evolutionary processes leading to the distribution and phenotypes of species and populations is a central goal in biogeography. Divergent selection along environmental gradients may lead to phenotypic and genotypic differentiation, potentially resulting in reproductive isolation and speciation (Smith et al. 2011; Schneider et al. 1999; Ogden and Thorpe 2002; Mittelbach et al. 2007). Phenotypic divergence coupled with genetic differentiation is generally more likely to occur where physical barriers prevent gene flow between populations (Hendry and Taylor 2004; Nosil and Crespi 2004; Crispo et al. 2006). Consequently, species found in highly distinct environments allow for the study of patterns of phenotypic and genetic differentiation aimed at deciphering the common evolutionary forces leading to observed patterns of intraspecific diversity.

The Western honey bee *Apis mellifera* is such a possible model species. The intraspecific diversity and phylogeography of this species has been examined extensively (Engel 1999; Ruttner 1988). According to Whitfield et al. (2006) and Kotthoff et al. (2013) the species originated in Africa and had a huge native range extending from South Africa to Scandinavia and from the Iberian Peninsula to Central Asia. Humans later introduced it to the Americas, Australia, and East Asia.

In sub-Saharan Africa, where the bee populations are largely feral, observed levels of genetic differentiation between morphologically defined neighboring subspecies are generally low (with the exception of the unusual subspecies pair of the thelytokous *A. m. capensis* and *A. m. scutellata*, Neumann et al. 2011), probably due to the extreme degree of panmixia and large dispersal capacity of honey bee colonies (Franck et al. 2001). Virgin queens mate with tens of partners in drone congregation areas (DCA), which can contain thousands of males coming from over 200 colonies (Winston 1987; Baudry et al. 1998). The mating distance is large (up to 15 km in *A. m. mellifera*, Jensen et al. 2005) and the dispersal distances of reproductive swarms range from a few hundred meters to 10 km (Schneider 1995; Camazine et al. 1999; Seeley and Morse 1978). Absconding (colony movements to a new nest site without swarming) due to predation, parasite infestation, reduced food availability, or other adverse circumstances is frequent among African honey bee subspecies (Fletcher 1978; Schneider and Mcnally 1992). It has been estimated, based on engorgement and metabolic rates, that reproductive swarms and absconding colonies may move even much further than 10 km (>50 km, Otis et al. 1981).

**Figure 1:**
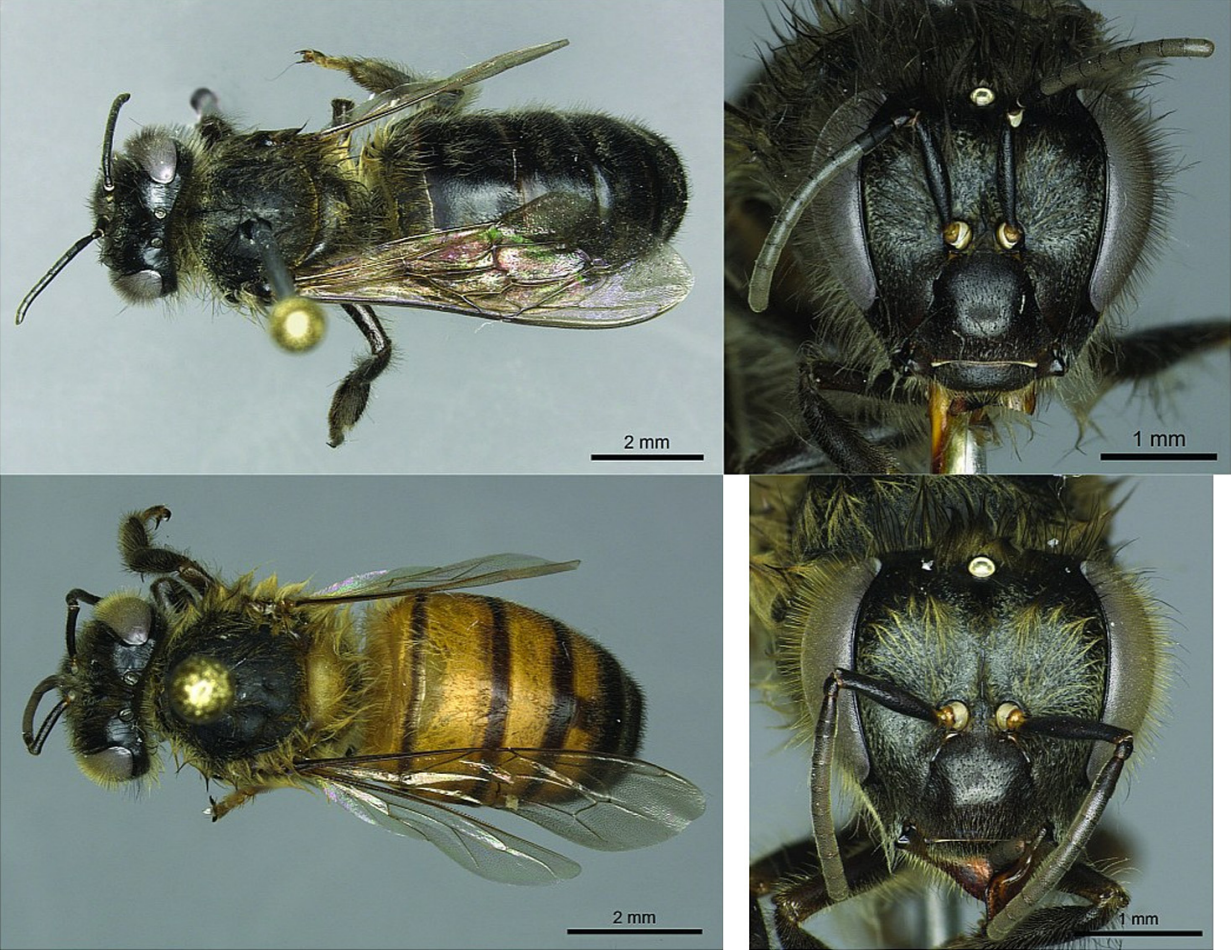
Morphology of Kenyan *Apis mellifera* worker bees. Upper row: Dorsal view of entire specimen and head in frontal view of typical individual from montane forest population at Mount Kenya (ANTWEB CASENT0249086). Lower row: Dorsal view of entire specimen and head in frontal view of typical individual from savanna population at Mount Kenya (ANTWEB CASENT0249088). Photographs are courtesy of April Nobile and ANTWEB at http://www.antweb.org.

Among the many extant African honey bee subspecies, *A. m. monticola* Smith 1961 represents a case of special interest because it shows a disjunct distribution in small “Islands” of montane forests across East Africa. Due to its special plate tectonic dynamics, East Africa has a highly complex topography with a large associated diversity in vegetation types (McClanahan and Young 1996, White 1983). The scattered high mountains (up to 5900 m), most of which are of volcanic origin, are characterized by three distinct vegetation belts (Bussmann 2006): montane forests (with or without a bamboo zone at their upper limits), subalpine heathlands (*Erica* bush), and an alpine zone. Savanna vegetation usually occurs in areas below 1000 m a.s.l. Today the forests on the various mountains are isolated from each other, cover very little land area and are endangered by economically attractive alternative uses such as agricultural conversion and timber exploitation (Gathaara 1999). The subspecies *A. m. monticola* has been found in these isolated montane forests above 2000 m a.s.l. in Kenya and Tanzania (Meixner et al. 1989, 1994, 2000; Ruttner 1988) but some authors have also assigned specimens collected in Burundi, Ethiopia, and Malawi to this subspecies (Franck et al. 2001; Ruttner 1988). The lower lying agriculture and savanna habitats surrounding the montane forests in Kenya and Tanzania are inhabited by *A. m. scutellata* Lepeletier 1836 which is widely distributed from eastern to southern Africa (Hepburn 1998; Ruttner 1988). Workers of *A. m. monticola* are somewhat larger and darker than those of *A. m. scutellata* (Ruttner 1988) (Fig. 1).

Two hypotheses seek to explain the distribution of these two subspecies in Kenya and Tanzania. The mountain refugia hypothesis (Meixner et al. 1989, 1994, 2000) proposes that *A. m. monticola* is a distinct lineage which evolved at an unspecified time in a high altitude forest area and had a large historical distribution during the last glacial maximum, between 21000 and 15000 years BP when forest vegetation shifted about 1000 m downslope (Osmaston 1989; Brühl 1997; Rucina et al. 2009). This lineage became isolated on the different mountains they currently inhabit due to climate change and associated changes in vegetation. Prezygotic mating barriers have been suggested to exist between other subspecies (Koeniger et al. 1989; Soland-Reckeweg et al. 2009; Oleksa et al. 2013) and may also occur in *A. m. monticola* to help maintain the morphological distinctness of their populations compared with *A. m. scutellata*. Data on mitochondrial DNA polymorphisms and allozyme variability seem to support the mountain refugia hypothesis (Meixner et al. 1994, 2000; Lind et al. 2010). On the other hand, the forest areas are very small relative to the mating and colony movement distances and so extensive gene flow and introgression between *A. m. monticola* and *A. m. scutellata* seems likely. Moreover, it remains unclear whether all the forests harboring *A. m. monticola* populations today such as Mount Kilimanjaro and Mount Kenya were really linked during the last glacial maximum (Brühl 1997). The alternative hypothesis is that the divergent phenotypes of honey bees in montane forests and lower lying agricultural and savanna areas are an example of phenotypic plasticity within a single bee lineage.

High gene flow between selective environments may favor the evolution of increased phenotypic plasticity over adaptive genetic divergence between populations (Crispo 2008; Lind et al. 2010). Pronounced phenotypic plasticity may likewise be selectively favored in honey bee populations in the East African region because of the documented life-history traits of the honey bee and the topographic heterogeneity of the region. Here, we examined populations in montane forests and nearby savanna regions at three mountain systems in Central Kenya. We employed morphometric analyses, mtDNA sequences and microsatellites to test the two hypotheses about the origin of extant *A. m. monticola* populations.

## Methods

### Honey bee samples

**Figure 2:**
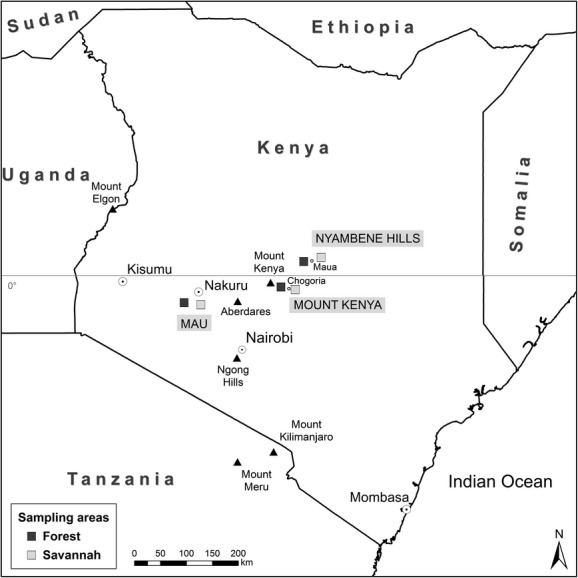
Map of study sites including places of sample collection. At each of the three mountain systems, Nyambene Hills, Mount Kenya, and Mau, we collected female *Apis mellifera* from a montane forest population and a corresponding savanna population nearby.

Samples of adult worker honeybees (Fig. 1) were collected from three montane forest areas above 2000 m altitude (Nyambene Hills, eastern slope of Mount Kenya, Eastern Mau Forest) and three nearby savanna areas in September 2009 (Fig. 2, Supplementary Table 1, Appendix S1). All the three mountain areas had been reported to harbor *A. m. monticola* Smith 1961, while the lowland areas surrounding the respective mountains are inhabited by *A. m. scutellata* Lepeletier 1836 (Meixner et al. 1994, 2000; Ruttner 1988). All our forest sampling sites support closed canopy forests. For many years, exploitation of the forests and their wildlife was not regulated in Kenya, which led to degradation, destruction, and fragmentation on a large scale (Beentje #ece3711-bib-1000^1990^; Bussmann #ece3711-bib-2000^1994^; Gathaara 1999). At the eastern slope of Mount Kenya our forest sampling site was located more than 8.5 km away from the forest edge. By contrast, even the largest remaining patch of the Nyambene Hills forest now has a diameter of less than 7 km and the forest strip where we worked in the Eastern Mau Forest was less than 8 km wide. Our sampling areas in “savanna habitat” either supported savanna vegetation (grass with sparse tree cover) or were used for smallholder agriculture with some trees. According to the information of local informants or our field assistants, none of these sites have been forested within the last 40 years. The positions and altitude of the colonies were determined with a GPS device (GARMIN®, model “Etrex Summit”, Garmin International, Olathe, KS) and are listed in Supplementary material (Supplementary Table 1, Appendix S1). The distance between the mountain area and the corresponding savanna area was between 21 and 24 km, and the difference in altitude between these areas was between 1000 m and 1300 m. The size of the area from which samples were taken varied from 2 to 5 km^2^ among the six collection sites (denoted in the following as populations). All six sampled populations are considered feral, as there are no reports of breeding efforts or successful introductions of foreign honey bees in these areas (see also Fletcher 1978).

### Morphological analyses

A total of 300 individuals representing 30 colonies (five from each of the six populations) were examined morphometrically (Supplementary Table 1, Appendix S2). We randomly selected 10 workers from each of these 30 colonies, removed the right hind leg for later genetic analyses, and dry mounted the specimens. Using a LEICA® MS 5 stereomicroscope (LEICA Microsystems GmbH, Wetzlar, Germany) and a pin-holding stage that allows endless rotations around the X, Y, and Z axes, we determined for each specimen:

HW: head width in frontal view (including eyes).

SL: length of the left antennal scape.

HTL: length of the left hind tibia.

Pigmentation – number of segments that are not completely dark among the following six segments: scutellum, tergit 1- tergit 5. In entirely dark specimens this value is zero (see exemplary specimens in Fig. 1).

For each colony, we calculated the mean values and used these data for principal component and discriminant function analyses. To test for linear relationships between these characters and altitude we calculated Pearson's *r* (using the data from all colonies from all six populations). These statistical analyses were carried out in STATISTICA 8 (StatSoft (Europe) GmbH, Hamburg, Germany). Voucher specimens from the six populations have been deposited in the National Museum (Invertebrate Zoology Section) in Nairobi.

### Molecular analyses

#### DNA extraction

We sampled between 9–10 colonies from each of the six localities (Mount Kenya Forest, Mount Kenya Savanna, Nyambene Hills Forest, Nyambene Hills Savanna, Mau Forest and Mau Savanna). For our smaller dataset (Supplementary Table 3, Appendix S2) we used one individual per colony (56 individuals total) for the STRUCTURE, Isolation by Distance (IBD) and genetic differentiation (F_st_ and Jost's D) analyses described below. We also obtained a larger dataset (Supplementary Table 4, Appendix S2), where we sampled additional individuals per colony (3–4), which were used for scoring the microsatellite loci, and to obtain an broader picture of the genetic divergence among the six populations. DNA extraction was done using the PureGene DNA extraction kit (Qiagen, Hilden, Germany), following the manufacturer's instructions.

#### PCR amplification

Nine polymorphic microsatellite loci (Estoup et al. 1995b) were scored for 56 individuals obtained for genetic analyses (Supplementary Table 3, Appendix S2): B124, A113, A24, A28, A88, A43, A007, A079, and A107 with variable annealing temperatures and Mg concentrations for optimal PCR amplification (Supplementary Table 5, Appendix S1). Two hundred and ninety-four individuals representing multiple individuals per colony were scored using microsatellites B124, A113, A24, A28, A88, and A43. PCR reactions were performed in 10 μL reaction volume, using Gotaq enzyme (Promega, Mannheim, Germany), following the manufacturers recommendations, and 1 μL of diluted genomic DNA. The forward primer was labeled at the 5'-end with one of three fluorescent dyes 6-FAM, JOE and ATTO, TAMRA and 2 μL of the resulting PCR mixture was added to 10 mL formamide and sent for genotyping at a local facility (BioMedical Research Center, Heinrich Heine University Düsseldorf and Cologne Centre of Genomics, CCG, University of Cologne, Germany). Mitochondrial DNA amplification of a 1104-bp fragment of the cytochrome oxidase gene (COI-CO II) intergenic region was carried out using primers E2 and H2 (Garnery et al. 1993), with Gotaq polymerase enzyme (Promega, Mannheim, Germany), following the manufacturers recommendations for 42 individuals representing 1 individual per colony, for selected colonies (Supplementary Table 2, Appendix S2). The resulting amplifications were verified on a 1% agarose gel and positive PCR amplifications were sent for direct sequencing (Eurofins MWG, Ebersberg, Germany).

#### Sequence analysis

Sequences were aligned manually using the program BIOEDIT version 7.0.9 (http://www.mbio.ncsv.edu/RNaseP/info/programs/BIOEDIT/bioedit.html; Hall 1999). All sequences and their alignments have been deposited in GenBank (dbGSS ID 37362734–37362775). We constructed haplotype networks using TCS v1.21 (Clement et al. 2000) to obtain a nonbifurcating perspective of relationships among haplotypes based on parsimony. We used 96% as probability connection limit with the important information of gaps (as 5th state) in the alignment.

### Microsatellite analyses

Microsatellite allele sizes were scored using an ABI 330 sequencer, which calculated allele sizes based on comparison of the lengths of the PCR products with those of the standard used during each run (Dye used: ROX). Microsatellite allele sizes were further scrutinized manually to detect null alleles and errors in allele scoring. Null alleles refer to the failure to amplify, via PCR, a specific microsatellite, due to mutations in the primer binding site, for example. We excluded all individuals where at least one null allele was detected. Within each colony, alleles differences of 1 bp were assumed to be a genotype scoring error and were rounded up to the next higher value (Amos et al. 2007). Microsatellite loci were tested for linkage disequilibrium (LD) for each pair of loci in each population and for conformation to Hardy–Weinberg Equilibrium (HWE) using ARLEQUIN v.3.5.1.2 (Excoffier and Lischer 2010). Genetic diversity was compared among different populations using various estimates. For each population we calculated the number of alleles (Na), expected (He) and observed (Ho) heterozygosity as implemented in GENALEX (Peakall and Smouse 2006).

### Genetic differentiation between honey bee populations

ARLEQUIN v.3.5.1.2 (Excoffier and Lischer 2010) was used to estimate basic population parameters, GENALEX was used to calculate observed and expected heterozygosities and unbiased estimates of gene diversity (Nei 1973) were calculated with GENEPOP 4.1 (Rousset 2008). Genotype frequencies were tested against the expectation of HWE and for evidence of LD between loci using Arlequin v.3.5.1.2, as the assumption of HWE and no LD need to be met for population structure analyses. Genetic differentiation among and within populations was quantified using F_st_ and Jost' D estimates (D_est_) (Jost 2008). F_st_ estimates were calculated using Arlequin software, and D_est_ was estimated using the online software SMOGD (Crawford 2010). Departure from mutation-drift equilibrium was tested using BOTTLENECK (Cornuet and Luikart 1997). Significance was assessed using the Sign and the Wilcoxon tests for heterozygosity excess, as implemented in BOTTLENECK. For this test, we assumed that each collecting region (Mount Kenya, Mau, and Nyambene Hills) harbors a single population. This program detects possible bottleneck events. This may occur if the *A. m. monticola* populations are the result of upwards migration from *A. m. scutellata* populations along the altitudinal gradient.

### Genetic differentiation over geographical distance

Population structure estimates such as those produced by Bayesian clustering approaches (as those implemented in the software STRUCTURE, Pritchard et al. (2000)) can be confounded when the population under study shows a strong IBD pattern (Frantz et al. 2009). We tested for patterns of IBD as implemented in SPAGeDI, (Hardy and Vekemans 2002) using the genetic relatedness between pairs of individuals for all colonies from each population as a function of geographical distance. Comparisons between colonies from all three forest sites were aimed at testing for evidence of a divergence pattern fitting with the refugia hypothesis. For comparisons involving forest versus savanna populations within each collecting site, we looked for evidences of vertical migration between these two subspecies. Comparisons between the three savanna populations were performed to detect an IBD pattern in the *A. m. scutellata* savanna population, which represents a population with an extensive range with no obvious barriers to gene flow.

### Bayesian clustering analyses

#### Population structure and admixture analyses

A Bayesian approach for clustering of individuals into putative populations (*k*) was used to estimate population structure using the software STRUCTURE v2.3 (Pritchard et al. 2000). STRUCTURE uses a model-based clustering approach to estimate a number of populations (*K*) and to assign individuals to any of *K* populations, using the admixture model with correlated allele frequencies and 1,000,0000 iterations, with a burn-in of 2,00,000. We tested a range of *k* values of 1–12 populations, and for each *k* we ran this STRUCTURE routine three times to evaluate consistency. The method of Δ*K* (Evanno et al. 2005) was used to determine the value of *k* that best fit the data. We tested two different combinations of STRUCTURE settings, using different prior assumptions (by means of the LOCPRIOR option); first, not using the LOCPRIOR option and then using as prior each of the six sampling localities. The LOCPRIOR setting was used to detect hard-to-find structure, as this model has been shown to detect structure with lower levels of divergence, compared to other models and it is not supposed to be biased toward detecting false structure (Hubisz et al. 2009).

The program BOTTLENECK vs 1.2.02 (Cornuet and Luikart 1997) was used to detect evidence of population bottlenecks in the six populations under study. Bottleneck uses a sign test which relies on comparing the observed number of loci with heterozygosity excess compared to the number of these loci expected by chance under the infinite allele model. A second test employed in this program is based on allele frequency. Rare alleles are rapidly lost after a bottleneck event, changing the normal distribution pattern observed at equilibrium, which correspond to a “L-shaped” distribution. The Bottleneck software compares observed allele frequency in a population to the distribution expected under mutation-drift equilibrium. Bottlenecks events are detected by the absence of a characteristic “L-shaped” distribution of allele proportions. We used the program Geneclass2 (Piry et al. 2004) which uses simulation based on Monte Carlo algorithms to estimate the number of migrants, defined as individuals belonging to a given population other than the one where they were sampled. Alternatively, Geneclass2 assigns individuals as residents, meaning they belong to the population where they were sampled. The number of individuals determined as migrants are given by the likelihood-based test statistic implemented in Geneclass2 with a probability of *P* < 0.01.

## Results

### Morphological analyses

**Figure 3:**
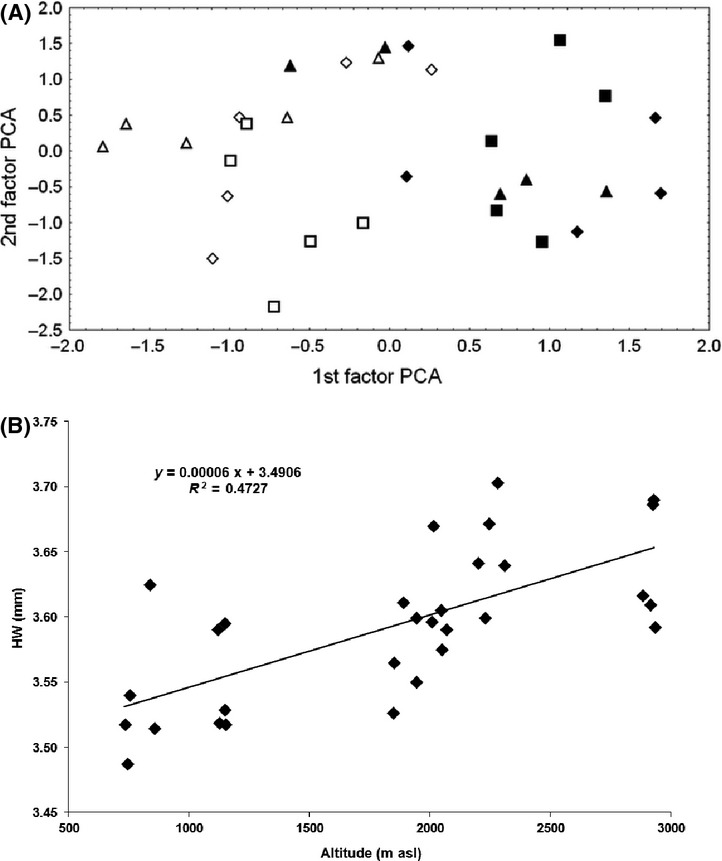
Analysis of morphometric characters from A. mellifera individuals. (A) Score plot from a principal component analysis (PCA) based on the colony means of four morphometric characters of five colonies each from the six study populations: ♦ Mount Kenya forest, ♢ Mount Kenya savanna, ▲ Nyambene Hills forest, ▵ Nyambene Hills savanna, ▪ Mau Forest, □ Mau savanna. (B) Linear relationship between mean head width and altitude for five colonies each from the six sampled populations (*r*^2^ = 0.473, *P* < 0.0001). The significant correlation suggests that differences in head morphology among workers are linked to altitude.

The morphometric data suggest that the colonies from mountain forest and savanna areas belong to two distinct groups (Fig. 3A). Although we used only four characters, the colonies from mountain forest and savanna areas are well separated in the discriminant function analysis (Wilks' Lambda = 0.3538; *F*_4,24_ = 11.4141, *P* < 0.0001). Interestingly, all four characters showed a linear correlation with altitude (Fig. 3B; *r*^2^ > 0.31, *P* < 0.002 in all cases), raising the question whether the observed pattern is due to the existence of two distinct morphological forms or rather represents continuous variation along an altitudinal gradient.

### Molecular analyses

In order to further test levels of differentiation between forest and savanna populations we used microsatellite data and mitochondrial DNA to evaluate genetic differentiation between populations. Overall our molecular analyses suggest there are no tangible differentiations between the two putative subspecies.

#### Biased data

Our tests for departure of HWE and LD suggest that there are only very small levels of both LD between loci and deviations from HWE for all populations in our small dataset, where we used one individual per colony. This dataset was used directly for subsequent genetic analyses.

#### Basic statistics

We first measured basic population levels parameters including unbiased estimates of gene diversity for each of the six populations on the small data set (*n* = 56) and obtained mean number of alleles (N_a_) that ranges from 8.11 to 9.78 and average gene diversity (expected heterozygosity, H_e_) ranging from 0.80 to 0.85 (Table 1). For all six populations, we obtained values of N_a_ and H_e_ not significantly different to each other (Table 1), which also holds, when forest against savanna populations were compared (e.g., forest H_e_ = 0.83 ± 0.02 versus savanna H_e_ = 0.83 ± 0.02). Summary statistic for each of the nine microsatellites separately are given in Table 2.

**Table 1:** Summary statistics of the nine microsatellite loci used in the analyses for six *Apis mellifera* populations and averages per forest and savanna regions

Population		N_a_	H_o_	H_e_	Population		N_a_	H_o_	H_e_
MF	Mean	9.78	0.71	0.85	MS	Mean	8.33	0.62	0.83
	SE	0.98	0.09	0.02		SE	0.47	0.1	0.02
MKF	Mean	8.11	0.63	0.80	MKS	Mean	8.67	0.67	0.83
	SE	0.94	0.09	0.04		SE	0.73	0.06	0.02
NHF	Mean	8.33	0.64	0.84	NHS	Mean	9.78	0.74	0.84
	SE	0.60	0.09	0.01		SE	0.97	0.06	0.02
Average	Mean	8.74	0.66	0.83	Average	Mean	8.93	0.68	0.83
	SE	0.84	0.09	0.02		SE	0.72	0.07	0.02

Number of alleles (N_a_), observed (H_o_) and expected (H_e_) Heterozygocity are given. MKF, Mount Kenya Forest; MKS, Mount Kenya Savanna; MF, Mau Forest; MS, Mau Savanna; NHF, Nyambene Hills Forest; NHS, Nyambene Hills Savanna.

**Table 2:** Summary statistics of the nine microsatellite loci used in the analyses of *Apis mellifera* populations

	N	N_a_	H_o_	H_e_		N	N_a_	H_o_	H_e_
Locus	A43				Locus	A88			
Mount Kenya forest	9	12	1	0.87	Mount Kenya forest	9	9	0.78	0.86
Mount Kenya savanna	9	10	0.67	0.88	Mount Kenya savanna	9	10	0.67	0.86
Nyambene hills forest	9	10	0.78	0.86	Nyambene hills forest	9	9	0.67	0.86
Nyambene hills savanna	10	11	0.80	0.88	Nyambene hills savanna	10	10	0.90	0.87
Mau forest	10	13	0.90	0.91	Mau forest	10	13	0.90	0.90
Mau savanna	9	9	0.89	0.83	Mau savanna	9	9	0.89	0.85
**Average**	**9.33**	**10.83**	**0.84**	**0.87**		**9.33**	**10.00**	**0.80**	**0.87**
Locus	A24				Locus	A113			
Mount Kenya forest	9	4	0.56	0.61	Mount Kenya forest	9	8	0.89	0.86
Mount Kenya savanna	9	7	0.89	0.73	Mount Kenya savanna	9	7	0.67	0.81
Nyambene hills forest	9	8	0.78	0.78	Nyambene hills forest	9	7	0.67	0.83
Nyambene hills savanna	10	6	0.7	0.76	Nyambene hills savanna	10	12	1	0.88
Mau forest	10	5	0.6	0.73	Mau forest	10	9	0.9	0.84
Mau savanna	9	6	0.67	0.65	Mau savanna	9	8	0.78	0.86
**Average**	**9.33**	**6.00**	**0.70**	**0.71**		**9.33**	**8.50**	**0.82**	**0.85**
Locus	A28				Locus	B124			
Mount Kenya forest	9	10	0.44	0.87	Mount Kenya forest	9	11	0.89	0.88
Mount Kenya savanna	9	9	0.89	0.85	Mount Kenya savanna	9	13	0.67	0.91
Nyambene hills forest	9	10	0.78	0.86	Nyambene hills forest	9	11	0.89	0.89
Nyambene hills savanna	10	9	0.70	0.85	Nyambene hills savanna	10	15	0.80	0.91
Mau forest	10	12	0.80	0.89	Mau forest	10	12	0.90	0.89
Mau savanna	9	8	0.78	0.84	Mau savanna	9	11	0.78	0.90
**Average**	**9.33**	**9.67**	**0.73**	**0.86**		**9.33**	**12.17**	**0.82**	**0.90**
Locus	A107				Locus	A079			
Mount Kenya forest	9	4	0.20	0.67	Mount Kenya forest	9	7	0.56	0.73
Mount Kenya savanna	9	7	0.286	0.796	Mount Kenya savanna	9	9	0.78	0.82
Nyambene hills forest	9	6	0	0.83	Nyambene hills forest	9	8	0.67	0.85
Nyambene hills savanna	10	8	0.37	0.78	Nyambene hills savanna	10	10	0.80	0.86
Mau forest	10	7	0.12	0.82	Mau forest	10	10	0.70	0.85
Mau savanna	9	7	0	0.84	Mau savanna	9	8	0.50	0.84
**Average**	**9.33**	**6.50**	**0.16**	**0.79**		**9.33**	**8.67**	**0.67**	**0.83**
Locus	A007				Locus	A007			
Mount Kenya forest	9	8	0.33	0.85	Nyambene hills savanna	10	6	0.60	0.76
Mount Kenya savanna	9	6	0.56	0.79	Mau forest	10	7	0.60	0.82
Nyambene hills forest	9	6	0.56	0.80	Mau savanna	9	9	0.33	0.85
**Average**	**9.07**	**6.70**	**0.32**	**0.81**		**9.47**	**7.73**	**0.54**	**0.82**

Number of samples (N), number of alleles (N_a_), observed (H_o_) and expected (H_e_) Heterozygocity are given.

#### Population genetic structure

**Figure 4:**
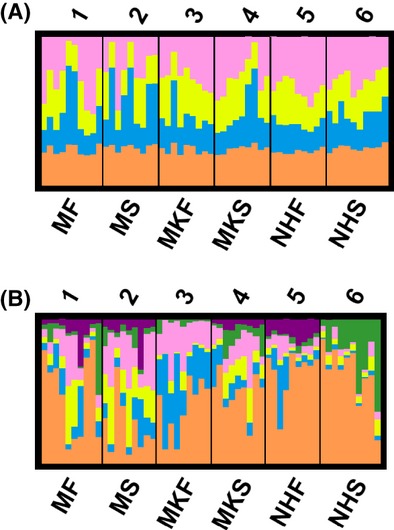
Results of Bayesian population structure analysis as implemented in the program STRUCTURE for the nine microsatellite data obtained on 56 females of *Apis mellifera*. Each vertical line represents the proportions of individual multilocus genotypes assigned to each of the *K* clusters estimated by the program. Figures show the resulting number of clusters that best fit the data for each of the two assumptions used, determined by the method of Evanno et al. (2005). (A) Not using the LOCPRIOR option, Ln'(*K* = 4): −2693.4. (B) Using as priors each of the six localities (MKF, MKS, MF, MS, NHF, NHS), Ln'(*K* = 6): −2855.525. Acronyms used: Mount Kenya Forest (MKF), Mount Kenya Savanna (MKS), Mau Forest (MF), Mau Savanna (MS), Nyambene Hills Forest (NHF), Nyambene Hills Savanna (NHS).

The results from the Bayesian cluster analysis used in STRUCTURE suggested that our six populations are best explained as belonging to either four (Fig. 4A) or six (Fig. 4B) different clusters, depending on the LOCPRIOR option used (see also Supplementary Table 2, Appendix S1). Overall, our analyses found no clear pattern of population division supporting the hypothesis of two genetically separated subspecies. Instead, we found that while some subdivision existed between *A. m. scutellata* and the putative *A. m. monticola* (Fig. 4A), the structure pattern is more a heterogenous one. When no prior was used each of the three localities (Mau, Nyambeme Hill, Mount Kenya) show a similar pattern of cluster for the two populations, whereas the amount of allele sharing is more equally distributed in Nyambeme Hills than in Mau (Fig. 4A, Supplementary Table 3, Appendix S1). Using the sampling populations (Mau Forest, Mau Savanna, Nyambene Hills Forest, Nyambene Hills Savanna, Mount Kenya Forest, and Mount Kenya Savanna, Fig. 4B) as prior (LOCPRIOR option in STRUCTURE) produced a slightly different outcome. The majority of individuals on Nyambeme Hills were preferably assigned to one cluster (orange, Fig. 4B, Supplementary Table 4, Appendix S1), whereas as the pattern for the other two regions still support a model in which these two supposed subspecies have extensive allele sharing.

#### Genetic differentiation between montane forest and savanna populations

**Figure 5:**
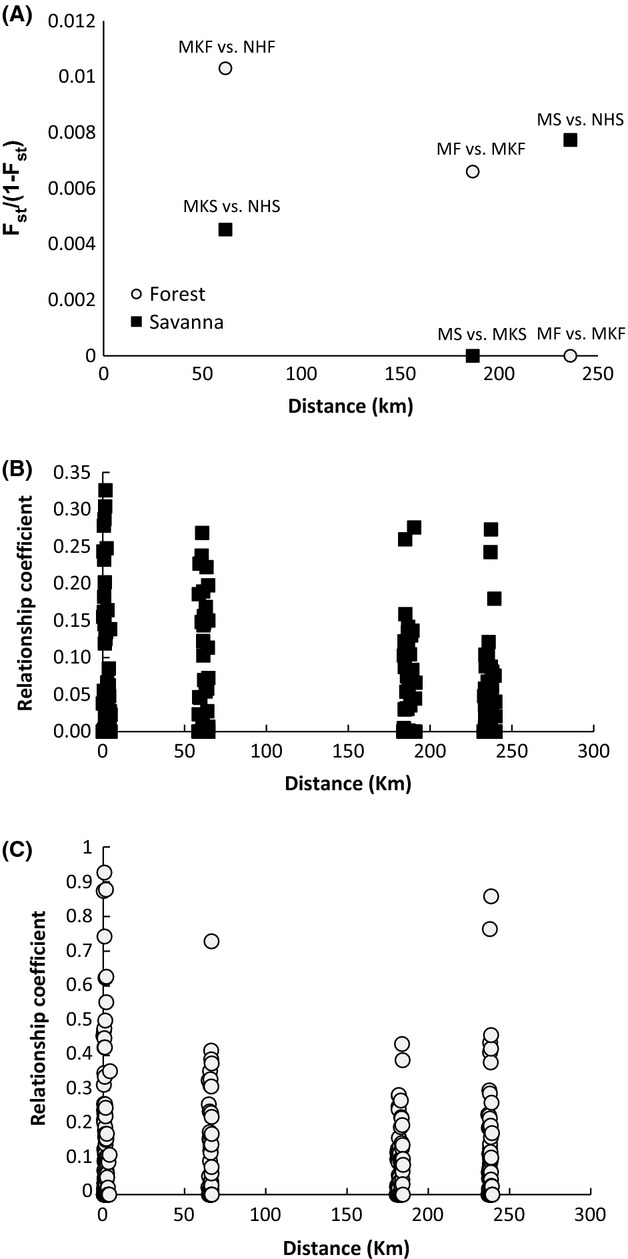
Genetic differentiation for pairwise comparisons of savanna and forest populations (A) using F_st_ statistics and of savanna (B) and forest (C) individuals using Queller and Goodnight's (1989) relationship coefficient, plotted against geographical distance in kilometers (km), as implemented in the software SPAGEDI. Results show no correlation between genetic and geographic distance. Acronyms used: Mount Kenya Savanna (MKS), Mau Savanna (MS), Nyambene Hills Savanna (NHS), Mount Kenya Forest (MKF), Mau Forest (MF), Nyambeme Hills Forest (NHF).

For the microsatellite dataset, we estimated differentiation using F_st_, with the program Arlequin, and D_est_ (Jost 2008), using the web-based program SMOGD (Crawford 2010). Table 3 show the values obtained for both statistics. We detected low levels of genetic differentiation using both F_st_ and Jost's D_st_ statistics, between high and low altitude populations. With either statistic, populations from high altitude sites showed low genetic differentiation, compared to any other high or low altitude locality. To evaluate genetic differentiation in pair of colonies over geographical distance we performed two tests: First the IBD test (Fig. 5), as implemented in SPAGeDI (Hardy and Vekemans 2002). We did not find evidence of a genetic pattern correlated with geographical distance for any combination of populations, whereas several migrants between localities were detected by the program Geneclass2 (Piry et al. 2004) (Supplementary Table 7, Appendix S1). Finally, the program BOTTLENECK detected no evidence of population bottlenecks in any of the six populations studied (Supplementary Table 8, Appendix S1).

**Table 3:** Genetic differentiation values obtained for Jost's D and F_st_ for pairwise comparisons between the six populations under study

	MF	MS	MKF	MKS	NHF	NHS
MF		0.316	0	0	0	0.14
MS	0.035		0.244	0.663	0.269	0.245
MKF	0.04	0.045		0.056	0.622	0.014
MKS	0.032	0.039	0.038		0.384	0
NHF	0.031	0.045	0.046	0.035		0.37
NHS	0.032	0.042	0.047	0.035	0.041	

All values are significant at *P* < 0.05. F_st_ values shown below diagonal, and Jost's D above diagonal. MKF, Mount Kenya Forest; MKS, Mount Kenya Savanna; MF, Mau Forest; MS, Mau Savanna; NHF, Nyambene Hills Forest; NHS, Nyambene Hills Savanna.

We also ran the same genetic analyses with the larger data set of 294 individuals comprising three to four individuals per colony. Even though the use of siblings may grossly overestimate population structure (Anderson and Dunham 2008; Rodriguez-Ramilo and Wang #ece3711-bib-7000^2012^), we did not obtain evidence suggesting strong genetic differentiation between montane forest and savanna populations and low genetic differentiation between montane forest populations as predicted by the mountain refugia hypothesis. The corresponding data are given in the Supporting Information, Appendix S2.

**Figure 6:**
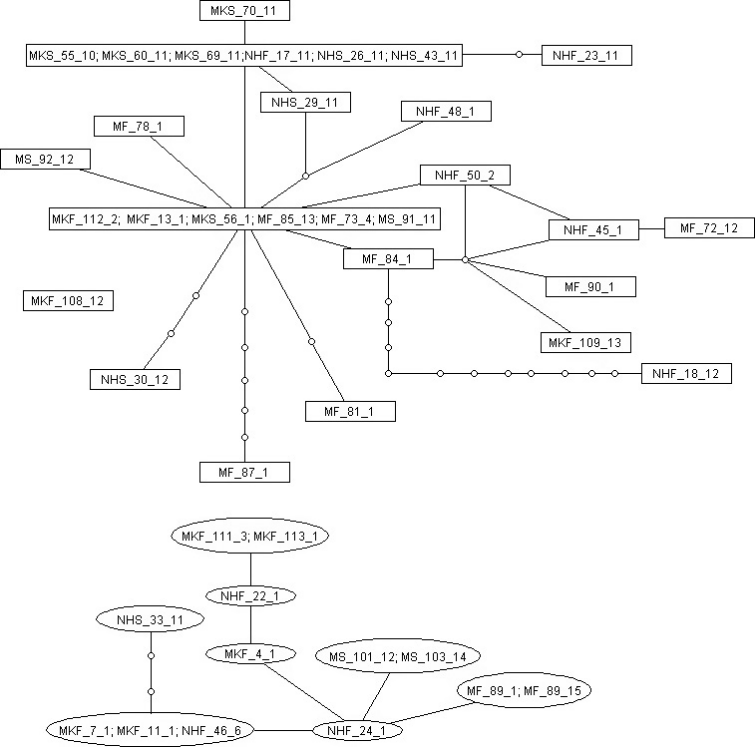
Haplotype network based on nucleotide substitution of the mitochondrial cytochrome oxidase gene (COI-CO II) intergenic region of 42 individuals representing montane forest (denoted as *monticola*) and savanna (denoted as *scutellata*) populations were used. 1104 bp alignment information of COI-COII intergenic region was used. Networks containing circled haplotypes are separated from the one in boxes basically by a 192 bp indel containing region. Abbreviations of the localities: MKF, Mount Kenya Forest; MKS, Mount Kenya Savanna; MF, Mau Forest; MS, Mau Savanna; NHF, Nyambene Hills Forest; NHS, Nyambene Hills Savanna.

Haplotype network analysis of mitochondrial sequence data of the COI-COII intergenic region revealed two haplotype networks (haplotypes in circles, Fig. 6) and one haplotype, supported by 18 sequences (large box), which can be connected to one singleton. After manual inspection, the sequences represented in boxes are differentiated from the rest by a 192 bp containing region, which is absent in the circled haplotypes. This analysis provided no differentiation of mitochondrial haplotypes according to their place of sampling, as most haplotypes are present in both forest and savanna localities.

## Discussion

Here, we document patterns of phenotypic variation in the widely distributed Western honey bee *Apis mellifera*, focusing on two subspecies from East Africa, *A. m. monticola* and *A. m. scutellata*. Our combined approach, measuring morphological and genetic parameters, sheds light on the level of differentiation between *A. m. monticola* and *A. m. scutellata* populations in Kenya.

### Morphological versus genetic differentiation of Kenyan honey bees

Honey bee subspecies have traditionally been classified on the basis of morphometric differences (Ruttner 1988). With the four morphometric characters we used, the colonies from high altitude montane forests are clearly distinguishable from those living in savanna habitats (Fig. 3A). These findings are in agreement with the reports by Meixner et al. (1989, 1994) for East African honey bees and with a study of bees from mountain systems across sub-saharan Africa (Hepburn et al. 2000). However, the morphological differences between colonies from montane forests and savanna areas may result from a linear correlation of the examined characters with altitude (Fig. 3B). Thus, using only morphometric data does not provide enough information to determine which of the current hypotheses about the origin of *A. m. monticola* populations is best supported.

Our genetic analyses show extensive sharing of mitochondrial alleles between the montane forest and savanna populations studied, as well as low levels of population differentiation between the two groups, based on microsatellite data. These results are in remarkable contrast with previous reports on these two putative subspecies in Kenya, which found corresponding morphological and genetic differentiation between them, supporting their taxonomic status (Meixner et al. 1994, 2000). Haplotype network analysis of mitochondrial DNA sequences provided no differentiation of the haplotypes according to their place of sampling, as most haplotypes are present in both forest and savanna localities (Fig. 6). Our F_st_ and D_est_ estimates from microsatellite data further supported this finding, showing low levels of differentiation between any of population pairs (Table 3). F_st_ and D_est_ values between forest populations are in the range of those observed between montane forest and savanna populations. This suggests that while all populations are to some extent differentiated from each other, populations belonging to the same habitat (forest or savanna) are not more similar to each other than to those in the other habitat type. As an interesting exception, the F_st_ and D_est_ values for MKF, NHF, and NHS are highest although closely located (Fig. 2), which may reflect altitudinal differentiation that needs to be further studied. F_st_ values obtained in this study are strikingly different from those obtained by Soland-Reckeweg et al. (2009) for comparisons between subspecies of *A. mellifera* in Switzerland, which are on average an order of magnitude larger than the F_st_ values we found. However, a study of the genetic differentiation among *A. mellifera* populations in Italy using microsatellites (Dall'Olio et al. 2007) provided F_st_ values between populations of *A. m. ligustica* similar to those found in our study. It should be noted, however, that the anthropogenic effect on honeybee population structure in Europe is much more pronounced than in Kenya. Consequently, naturally occurring gene flow among honeybee populations in Kenya most likely contributes to the low genetic differentiation we found.

Bayesian clustering analyses (Fig. 4) show that the analyzed populations of these two subspecies are not assigned to different clusters, as would be expected for separate populations. Instead, no individuals, whether from montane forests or savanna, were assigned to any one cluster with high probability. These results suggest extensive allele sharing between montane forests or savanna populations. Including the sampling localities as prior information has been reported to provide a more sensitive assessment of population structure (Ostrowski et al. 2006), however, only a slight improvement in the degree of structure was detected for some populations (Fig. 4B).

Combining the morphological data, which show clear differences between the two groups of populations and were significantly correlated with altitude (Fig. 3A and B), with the molecular data showing levels of population differentiation comparable only to previous reports for within-subspecies variation of honey bees, support the hypothesis of phenotypic plasticity, and the single lineage hypothesis for montane forest and savanna populations.

### The mountain refugia hypothesis revisited

Although East Africa has been described as “mosaic of spatial and temporal refugia” by Lorenzen et al. (2012), clear evidence supporting refugia models that explain current distributions of very young subspecies, such as from the last 25,000 thousand years, are rare. In order to explain the current distribution of *A. m. monticola*, the mountain refugia hypothesis was favored by Meixner et al. (2000). The main conclusion of this work was based on just two haplotypes, shared by mountain colonies, and one haplotype found for savanna colonies. A key prediction of this hypothesis is that populations from *A. m. monticola*, found in different montane localities, should exhibit low genetic differentiation among themselves, as a reflection of their shared genetic past. We would also expect to find lower similarity between montane forest and close-by savanna populations, compared to levels between montane forest populations, as expected if they indeed belong to different subspecies. However, several honey bee life-history traits such as their high degree of panmixia (Franck et al. 2000), large mating range, long dispersal distances and the historical and recent reduction in size of montane forests through logging, make the refugia scenario unlikely. Our study provides a more comprehensive analysis of the genetic background of these two honey bee subspecies, and shows no support for a significant differentiation between the two currently accepted subspecies, both at the level of mitochondrial DNA sequences and microsatellites. These results are similar to those of Franck et al. (2001), who found little mitochondrial DNA differentiation among African honey bee subspecies over large geographical scales. Likewise, microsatellite data did not support the existence of two different clusters or populations, which, coupled with the morphological divergence found, give stronger support for a phenotypic plasticity scenario.

### Phenotypic plasticity in Kenyan honey bees

Phenotypic plasticity is defined as the ability of a genotype to produce different phenotypes, depending on environmental conditions such as food, ambient light, temperature, and other ecological characteristics. From an evolutionary perspective, phenotypic plasticity can be adaptive because of past or ongoing selection regimens, allowing organisms to quickly respond to changing environmental conditions or to different conditions encountered after dispersal (Agrawal 2001; de Jong 2005; Nussey et al. 2007; Charmantier et al. 2008), which include a plastic response of the transcriptome, as found in Drosophila (Zhou et al. 2012). Here, we propose that phenotypic plasticity represents the most likely alternative to explain the phenotypic divergence and evolutionary history of montane forest and savanna honey bee populations. Phenotypic plasticity concerning melanisms and body size has been studied intensively in several insect species, where individuals from high altitude or latitude sites (representing colder climates), are darker and bigger than individuals from warmer climates (Bergmann's rule) (Clusella-Trullas et al. 2007). A strong positive correlation between dark pigmentation and high altitude has been reported in several insect groups, such as in flies (Pool and Aquadro 2007), beetles (Brakefield and Willmer 1985), and butterflies (Ellers and Boggs 2002). Two of the key differences between montane forest and savanna honeybee populations are their coloration and size (Fig. 3A). The presence of strong gene flow, for which we found evidence in our molecular data (Table 3, Fig. 4), can favor selection for pronounced phenotypic plasticity (Crispo 2008). Our proposed scenario of phenotypic plasticity could be tested in translocation experiments in which one would reciprocally transplant colonies to other habitat types (savanna colonies to montane forest habitats and vice versa) and examine the phenotype of the workers reared in the old and new environments. Savanna colonies translocated to montane forest habitat would be expected to produce workers with montane forest phenotype and vice versa. As an additional outcome of our study, we propose to critically examine the taxonomic status of *A. m. monticola*. In order to address the question whether *A. m. monticola* needs to be synonymized, it will be necessary to examine samples of *A. m. monticola* from their type locality, Mount Kilimanjaro. Furthermore, it would be worthwhile to focus on the closely related subspecies *A. m. litorea* which occurs at lower altitudes at the East African coast as it cannot be excluded that all these three “subspecies” represent morphological variants of a single, highly plastic group within the East African region.

## Biosketch

One of the senior author's main interest is to understand how biodiversity is related to changing environmental conditions and affects population genetic structure in social insects. For further information visit http://www.popbee.uni-koeln.de.

## Supporting Information

**Appendix S1.** Additional tables providing details on the individuals of *Apis mellifera* and their genetic relationship.

**Appendix S2.** Additional data providing details on genetic analysis of *Apis mellifera* populations.

## References

[b1] Agrawal AA (2001). Phenotypic plasticity in the interactions and evolution of species. Science.

[b2] Amos W, Hoffman JI, Frodsham A, Zhang L, Best S, Hill AVS (2007). Automated binning of microsatellite alleles: problems and solutions. Mol. Ecol. Notes.

[b3] Anderson EC, Dunham KK (2008). The influence of family groups on inferences made with the program structure. Mol. Ecol. Resour.

[b5] Baudry E, Solignac M, Garnery L, Gries M, Cornuet JM, Koeniger N (1998). Relatedness among honeybees (*Apis mellifera*) of a drone congregation area. Proc. R. Soc. Lond. B.

[b1000] Beentje H (1990). The forests of Kenya. Mitt. Inst. Allg. Bot. Hamburg.

[b7] Brakefield PM, Willmer PG (1985). The basis of thermal melanism in the ladybird Adalia bipunctata: differences in reflectance and thermal properties between the morphs. Heredity.

[b8] Brühl CA (1997). Flightless insects: a test case for historical relationships of African mountains. J. Biogeogr.

[b2000] Bussmann RW (1994).

[b9] Bussmann R (2006). Vegetation zonation and nomenclature of African mountains – an overview. Lyonia.

[b10] Camazine S, Visscher PK, Finley J, Vetter RS (1999). Househunting by honey bee swarms: collective decisions and individual behaviors. Insectes Soc.

[b11] Charmantier A, McCleery RH, Cole LR, Perrins C, Kruuk LE, Sheldon BC (2008). Adaptive phenotypic plasticity in response to climate change in a wild bird population. Science.

[b12] Clement M, Posada D, Crandall KA (2000). TCS: a computer program to estimate gene genealogies. Mol. Ecol.

[b13] Clusella-Trullas S, Spotila JH, van Wyk JR (2007). Thermal melanism in ectotherms. J. Therm. Biol.

[b14] Cornuet JM, Luikart G (1997). Description and power analysis of two tests for detecting recent population bottlenecks from allele frequency data. Genetics.

[b15] Crawford NG (2010). SMOGD: software for the measurement of genetic diversity. Mol. Ecol. Resour.

[b16] Crispo E (2008). Modifying effects of phenotypic plasticity on interactions among natural selection, adaptation and gene flow. J. Evol. Biol.

[b17] Crispo E, Bentzen P, Reznick DN, Kinnison MT, Hendry AP (2006). The relative influence of natural selection and geography on gene flow in guppies. Mol. Ecol.

[b18] Dall'Olio R, Marino A, Lodesani M, Moritz R (2007). Genetic characterization of Italian honeybees, *Apis mellifera ligustica*, based on microsatellite DNA polymorphisms. Apidologie.

[b19] Ellers J, Boggs CL (2002). The evolution of wing color in *Colias* butterflies: heritability, sex linkage, and population divergence. Evolution.

[b20] Engel MS (1999). The taxonomy of recent and fossil honey bees (Hymenoptera: Apidae, Apis). J. Hymenopteran Res.

[b21] Estoup A, Scholl A, Pouvreau A, Solignac M (1995b). Monoandry and polyandry in bumble bees (Hymenoptera; Bombinae) as evidenced by highly variable microsatellites. Mol. Ecol.

[b22] Evanno G, Regnaut S, Goudet J (2005). Detecting the number of clusters of individuals using the software structure: a simulation study. Mol. Ecol.

[b23] Excoffier L, Lischer HEL (2010). Arlequin suite ver 3.5: A new series of programs to perform population genetics analyses under Linux and Windows. Mol. Ecol. Resour.

[b24] Fletcher DJC (1978). The African honey bee, *Apis mellifera adansonii*, in Africa. Annu. Rev. Entomol.

[b26] Franck P, Koeniger N, Lahner G, Crewe RM, Solignac M (2000). Evolution of extreme polyandry: an estimate of mating frequency in two African honeybee subspecies, *Apis mellifera monticola* and *A. m. scutellata*. Insectes Soc.

[b27] Franck P, Garnery L, Loiseau A, Oldroyd BP, Hepburn HR, Solignac M (2001). Genetic diversity of the honeybee in Africa: microsatellite and mitochondrial data. Heredity.

[b28] Frantz AC, Cellina S, Krier A, Schley L, Burke T (2009). Using spatial Bayesian methods to determine the genetic structure of a continuously distributed population: clusters or isolation by distance?. J. Appl. Ecol.

[b29] Garnery L, Solignac M, Celebrano G, Cornuet JM (1993). A simple test using restricted PCR-amplified mitochondrial DNA to study the genetic structure of *Apis mellifera* L. Experientia.

[b30] Gathaara G (1999). Aerial survey of the destruction of Mt Kenya.

[b31] Hall TA (1999). BioEdit: a user-friendly biological sequence alignment editor and analysis program for Windows 95/98/NT. Nucleid Acids Symp. Ser.

[b32] Hardy OJ, Vekemans X (2002). SPAGeDi: a versatile computer program to analyse spatial genetic structure at the individual or population levels. Mol. Ecol. Notes.

[b33] Hendry AP, Taylor EB (2004). How much of the variation in adaptive divergence can be explained by gene flow? An evaluation using lake-stream stickleback pairs. Evolution.

[b34] Hepburn HR (1998). Honeybees of Africa Springer.

[b35] Hepburn HR, Radloff SE, Oghiake S (2000). Mountain honeybees of Africa. Apidologie.

[b36] Hubisz MJD, Falush D, Stephens M, Pritchard JK (2009). Inferring weak population structure with the assistance of sample group information. Mol. Ecol. Resour.

[b37] Jensen AB, Palmer KA, Boomsma JJ, Pedersen BV (2005). Varying degrees of *Apis mellifera ligustica* introgression in protected populations of the black honeybee, *Apis mellifera mellifera*, in northwest Europe. Mol. Ecol.

[b38] de Jong G (2005). Evolution of phenotypic plasticity: patterns of plasticity and the emergence of ecotypes. New Phytol.

[b39] Jost L (2008). G(ST) and its relatives do not measure differentiation. Mol. Ecol.

[b40] Koeniger G, Koeniger N, Pechhacker H, Ruttner F, Berg S (1989). Assortative mating in a mixed population of European honeybees, *Apis mellifera ligustica* and *Apis mellifera carnica*. Insectes Soc.

[b42] Kotthoff U, Wappler T, Engel MS (2013). Greater past disparity and diversity hint at ancient migrations of European honey bee lineages into Africa and Asia. J. Biogeogr.

[b43] Lind MI, Ingvarsson PK, Johansson H, Hall D, Johansson F (2010). Gene flow and selection on phenotypic plasticity in an island system of *Rana temporaria*. Evolution.

[b44] Lorenzen ED, Heller R, Siegismund HR (2012). Comparative phylogeography of African savannah ungulates1. Mol. Ecol.

[b45] McClanahan TR, Young TP (1996). East African ecosystems and their conservation.

[b46] Meixner M, Ruttner F, Koeniger N, Koeniger G (1989). The mountain bees of the Kilimanjaro region and their relationship to neighbouring bee populations. Apidologie.

[b47] Meixner MD, Sheppard WS, Dietz A, Krell R (1994). Morphological and allozyme variability in honey bees from Kenya. Apidologie.

[b48] Meixner MD, Arias MC, Sheppard WS (2000). Mitochondrial DNA polymorphisms in honey bee subspecies from Kenya. Apidologie.

[b49] Mittelbach GG, Schemske DW, Cornell HV, Allen AP, Brown JM, Bush MB (2007). Evolution and the latitudinal diversity gradient: speciation, extinction and biogeography. Ecol. Lett.

[b50] Nei M (1973). Analysis of gene diversity in subdivided populations. Proc. Natl Acad. Sci. USA.

[b51] Neumann P, Härtel S, Kryger P, Crewe RM, Moritz RF (2011). Reproductive division of labour and thelytoky result in sympatric barriers to gene flow in honeybees (*Apis mellifera* L.). J. Evol. Biol.

[b52] Nosil P, Crespi B (2004). Does gene flow constrain adaptive divergence or vice versa? A test using ecomorphology and sexual isolation in *Timema cristinae* walking-sticks. Evolution.

[b53] Nussey DH, Wilson AJ, Brommer JE (2007). The evolutionary ecology of individual phenotypic plasticity in wild populations. J. Evol. Biol.

[b54] Ogden R, Thorpe RS (2002). Molecular evidence for ecological speciation in tropical habitats. Proc. Natl Acad. Sci.

[b55] Oleksa A, Wilde J, Tofiliski A, Chybicki IJ (2013). Partial reproductive isolation between European subspecies of honey bees. Apidologie.

[b56] Osmaston HA (1989). Glaciers, glaciations and equilibrium line altitudes on the Ruwenzori.

[b57] Ostrowski M, David J, Santoni S, McKhann H, Reboud X, Le Corre V (2006). Evidence for a large-scale population structure among accessions of Arabidopsis thaliana: possible causes and consequences for the distribution of linkage disequilibrium. Mol. Ecol.

[b58] Otis GW, Winston ML, Taylor OR (1981). Engorgement and dispersal of Africanized honeybee swarms. J. Apic. Res.

[b59] Peakall R, Smouse PE (2006). GENALEX 6: genetic analysis in Excel. Population genetic software for teaching and research. Mol. Ecol. Notes.

[b60] Piry S, Alapetite A, Cornuet JM, Paetkau D, Baudouin L, Estoup E (2004). GENECLASS2: A software for genetic assignment and first-generation migrant detection. J. Heredity.

[b61] Pool JE, Aquadro CF (2007). The genetic basis of adaptive pigmentation variation in Drosophila melanogaster. Mol. Ecol.

[b62] Pritchard JK, Stephens P, Donnelly P (2000). Inference of population structure using multilocus genotype data. Genetics.

[b7000] Rodriguez-Ramilo ST, Wang J (2012). The effect of close relatives on unsupervised Bayesian clustering algorithms in population genetic structure analysis. Mol. Ecol. Resour.

[b63] Rousset F (2008). Genepop'007: a complete reimplementation of the Genepop software for Windows and Linux. Mol. Ecol. Resour.

[b64] Rucina SM, Muiruri VM, Kinyanjui RN, McGuiness K, Marchant R (2009). Late Quaternary vegetation and fire dynamics on Mount Kenya. Palaeogeogr. Palaeoclimatol. Palaeoecol.

[b65] Ruttner F (1988). Biogeography and Taxonomy of Honeybees.

[b66] Schneider SS (1995). Swarm movement patterns inferred from waggle dance activity of the neotropical African honey bee in Costa Rica. Apidologie.

[b67] Schneider SS, Mcnally LC (1992). Factors influencing seasonal absconding in colonies of the African honey bee: *Apis mellifera scutellata*. Insectes Soc.

[b68] Schneider C, Smith TB, Larison B, Moritz C (1999). A test of alternative models of diversification in tropical rainforests: ecological gradients vs. rainforest refugia. Proc. Natl Acad. Sci. USA.

[b69] Seeley TD, Morse RA (1978). Nest site selection by the honey bee, *Apis mellifera*. Insectes Soc.

[b70] Smith FG (1961). The races of honeybees in Africa. Bee World.

[b71] Smith TB, Thomassen HA, Freedman AH, Sehgal RNM, Buehrmann W, Saatchi S (2011). Patterns of divergence in the olive sunbird *Cyanomitra olivacea* (Aves: Nectariniidae) across the African rainforest-savanna ecotone. Biol. J. Linn. Soc.

[b72] Soland-Reckeweg G, Heckel G, Neumann P, Fluri P, Excoffier L (2009). Gene flow in admixed populations and implications for the conservation of the Western honeybee, Apis mellifera. J. Insect Conserv.

[b73] Tamura K, Peterson D, Peterson N, Stecher G, Nei M, Kumar M (2011). MEGA5: molecular evolutionary genetics analysis using maximum likelihood, evolutionary distance, and maximum parsimony methods. Mol. Biol. Evol.

[b74] White F (1983). The vegetation of Africa A descriptive memoir to accompany the UNESCO/AETFAT/UNSO Vegetation Map of Africa (3 Plates, Northwestern Africa, Northeastern Africa, and Southern Africa, 1:5,000,000).

[b75] Whitfield CW, Behura SK, Berlocher SH, Clark AG, Johnston JS, Sheppard WS (2006). Thrice out of Africa: ancient and recent expansions of the honey bee, *Apis mellifera*. Science.

[b76] Winston ML (1987). The biology of the honey bee.

[b77] Zhou S, Campbell TG, Stone EA, Mackay TFC, Anholt RRH (2012). Phenotypic plasticity of the Drosophila transcriptome. PLoS Genet.

