# Supplementary material for: Distinct subspecies or phenotypic plasticity? Genetic and morphological differentiation of mountain honey bees in East Africa
Source: Ecol Evol. 2013 Aug 6;3(10):3204–18. doi: 10.1002/ece3.711 (PMC3797471; doi:10.1002/ece3.711)
Supplement: Supplementary file 1 [file ece30003-3204-SD1.docx]

**Supporting Information**

**Appendix S 1: pages 1- 8**

**Appendix S 2: pages 9 - 21**

**Supplementary Table 1.** List of sample localities used for this study, their coordinates and altitude (each given by one representative colony).

| Locality | Altitude (m) | Coordinates | |
| --- | --- | --- | --- |
| Mount Kenya Forest | 2389 | S 0° 11.813' | E 37° 28.929' |
| Mount Kenya Savanna | 1247 | S 0° 14.413' | E 37° 46.641' |
| Nyambene Hills Forest | 2066 | N 0° 13.697' | E 37° 53.743' |
| Nyambene Hills Savanna | 779 | N 0° 17.461' | E 38° 06.156' |
| Mau Forest | 2880 | S 0° 30.363' | E 35° 53.070' |
| Mau Savanna | 1884 | S 0° 31.273' | E 36° 05.472' |

**Supplementary Table 2**

Output for statistics calculated for STRUCTURE results using the Evano et al. (2005) method for determining the optimal number of clusters (in yellow), as implemented in the online application Structure Harvester (http://taylor0.biology.ucla.edu/structureHarvester/). A. Results for model not using the LOCPRIOR option. B. Results using as priors each of the six collecting localities (MKF, MKS, MF, MS, NHF, NHS).

| A. |  |  |  |  |  |
| --- | --- | --- | --- | --- | --- |
| #K | Mean LnP(K) | Stdev LnP(K) | Ln'(K) | \|Ln''(K)\| | Delta K |
|  |  |  |  |  |  |
| **2** | -3079.2 | 277.1258 | NA | NA | NA |
| **3** | -2737.125 | 56.6802 | 342.075 | 298.35 | 5.26374 |
| **4** | -2693.4 | 20.0543 | 43.725 | 158.6 | 7.908544 |
| **5** | -2808.275 | 51.639 | 114.875 | 98.525 | 1.907959 |
| **6** | -2824.625 | 46.564 | -16.35 | 125.775 | 2.701119 |
| **7** | -2966.75 | 95.7079 | 142.125 | 56.225 | 0.587465 |
| **8** | -3052.65 | 142.107 | -85.9 | 149.425 | 1.051496 |
| **9** | -2989.125 | 85.5125 | 63.525 | 151.4 | 1.770501 |

B.

| #K | Mean LnP(K) | Stdev LnP(K) | Ln'(K) | \|Ln''(K)\| | Delta K |
| --- | --- | --- | --- | --- | --- |
|  |  |  |  |  |  |
| **2** | -2664.475 | 15.4836 | NA | NA | NA |
| **3** | -2686.825 | 31.3836 | -22.35 | 59.425 | 1.893506 |
| **4** | -2768.6 | 51.5659 | -81.775 | 193.025 | 3.743265 |
| **5** | -3043.4 | 107.5231 | -274.8 | 462.675 | 4.303028 |
| **6** | -2855.525 | 68.1351 | 187.875 | 581.9 | 8.540388 |
| **7** | -3249.55 | 171.9688 | 394.025 | 606.175 | 3.524913 |
| **8** | -3037.4 | 349.4328 | 212.15 | 117.275 | 0.335615 |
| **9** | -2942.525 | 423.5128 | 94.875 | 256.375 | 0.605354 |
|  |  |  |  |  |  |

**Supplementary Table 3**

Individual proportion of cluster assignment for STRUCTURE analysis presented in Figure 4a.

| **Individual** | Cluster 1 | Cluster 2 | Cluster 3 | Cluster 4 | **Individual** | Cluster 1 | Cluster 2 | Cluster 3 | Cluster 4 |
| --- | --- | --- | --- | --- | --- | --- | --- | --- | --- |
| **N071_4** | 0.224 | 0.15 | 0.18 | 0.446 | **N014_1** | 0.207 | 0.149 | 0.219 | 0.425 |
| **N072_1** | 0.272 | 0.189 | 0.319 | 0.22 | **N016_5** | 0.211 | 0.155 | 0.237 | 0.397 |
| **N073_1** | 0.214 | 0.16 | 0.274 | 0.351 | **N055_15** | 0.25 | 0.154 | 0.32 | 0.275 |
| **N078_2** | 0.226 | 0.263 | 0.285 | 0.226 | **N056_13** | 0.253 | 0.187 | 0.28 | 0.28 |
| **N081_2** | 0.276 | 0.531 | 0.168 | 0.025 | **N060_13** | 0.264 | 0.202 | 0.302 | 0.232 |
| **N084_4** | 0.273 | 0.497 | 0.178 | 0.052 | **N062_5** | 0.26 | 0.423 | 0.17 | 0.146 |
| **N085_3** | 0.212 | 0.168 | 0.324 | 0.296 | **N064_1** | 0.289 | 0.5 | 0.176 | 0.035 |
| **N087_4** | 0.204 | 0.135 | 0.167 | 0.494 | **N069_14** | 0.242 | 0.216 | 0.183 | 0.36 |
| **N089_13** | 0.212 | 0.141 | 0.127 | 0.52 | **N070_11** | 0.259 | 0.163 | 0.315 | 0.263 |
| **N090_4** | 0.283 | 0.252 | 0.253 | 0.212 | **N017_12** | 0.224 | 0.25 | 0.162 | 0.363 |
| **N091_13** | 0.263 | 0.182 | 0.319 | 0.236 | **N018_13** | 0.227 | 0.185 | 0.289 | 0.299 |
| **N092_15** | 0.274 | 0.522 | 0.172 | 0.032 | **N022_15** | 0.213 | 0.193 | 0.333 | 0.26 |
| **N093_5** | 0.221 | 0.152 | 0.13 | 0.496 | **N023_15** | 0.213 | 0.201 | 0.309 | 0.277 |
| **N094_1** | 0.264 | 0.348 | 0.168 | 0.22 | **N024_5** | 0.242 | 0.171 | 0.205 | 0.381 |
| **N098_4** | 0.274 | 0.521 | 0.174 | 0.031 | **N045_3** | 0.231 | 0.147 | 0.224 | 0.397 |
| **N101_12** | 0.25 | 0.166 | 0.309 | 0.275 | **N046_12** | 0.22 | 0.142 | 0.168 | 0.471 |
| **N103_14** | 0.217 | 0.171 | 0.333 | 0.278 | **N048_2** | 0.239 | 0.161 | 0.227 | 0.373 |
| **N104_14** | 0.27 | 0.416 | 0.188 | 0.126 | **N50_5** | 0.262 | 0.194 | 0.222 | 0.322 |
| **N105_5** | 0.279 | 0.409 | 0.191 | 0.121 | **N25_5** | 0.219 | 0.283 | 0.143 | 0.354 |
| **N04_2** | 0.253 | 0.199 | 0.276 | 0.272 | **N26_13** | 0.253 | 0.167 | 0.3 | 0.28 |
| **N07_3** | 0.214 | 0.189 | 0.343 | 0.255 | **N029_11** | 0.27 | 0.302 | 0.178 | 0.25 |
| **N011_2** | 0.29 | 0.421 | 0.192 | 0.097 | **N030_12** | 0.269 | 0.182 | 0.324 | 0.225 |
| **N013_2** | 0.207 | 0.185 | 0.347 | 0.26 | **N033_14** | 0.244 | 0.183 | 0.169 | 0.404 |
| **N108_14** | 0.273 | 0.21 | 0.312 | 0.205 | **N034_4** | 0.235 | 0.141 | 0.27 | 0.354 |
| **N109_13** | 0.269 | 0.198 | 0.25 | 0.283 | **N037_3** | 0.258 | 0.241 | 0.217 | 0.284 |
| **N111_14** | 0.21 | 0.174 | 0.301 | 0.315 | **N038_2** | 0.254 | 0.244 | 0.207 | 0.296 |
| **N112_13** | 0.231 | 0.206 | 0.204 | 0.36 | **N041_1** | 0.26 | 0.239 | 0.282 | 0.22 |
| **N113_13** | 0.215 | 0.198 | 0.212 | 0.375 | **N043_13** | 0.293 | 0.309 | 0.195 | 0.203 |

**Supplementary Table 4**

Individual proportion of cluster assignment for STRUCTURE analysis presented in Figure 4b.

| **Individual** | Cluster 1 | Cluster 2 | Cluster 3 | Cluster 4 | Cluster 5 | Cluster 6 | **Individual** | Cluster 1 | Cluster 2 | Cluster 3 | Cluster 4 | Cluster 5 | Cluster 6 |
| --- | --- | --- | --- | --- | --- | --- | --- | --- | --- | --- | --- | --- | --- |
| **N071_4** | 0.79 | 0.05 | 0.04 | 0.053 | 0.034 | 0.033 | **N014_1** | 0.533 | 0.263 | 0.028 | 0.136 | 0.015 | 0.025 |
| **N072_1** | 0.634 | 0.052 | 0.076 | 0.082 | 0.131 | 0.026 | **N016_5** | 0.688 | 0.138 | 0.041 | 0.076 | 0.026 | 0.031 |
| **N073_1** | 0.663 | 0.172 | 0.033 | 0.071 | 0.034 | 0.027 | **N055_15** | 0.392 | 0.06 | 0.179 | 0.1 | 0.201 | 0.069 |
| **N078_2** | 0.482 | 0.178 | 0.09 | 0.187 | 0.027 | 0.037 | **N056_13** | 0.444 | 0.113 | 0.126 | 0.139 | 0.105 | 0.073 |
| **N081_2** | 0.096 | 0.039 | 0.405 | 0.347 | 0.022 | 0.091 | **N060_13** | 0.514 | 0.099 | 0.11 | 0.144 | 0.105 | 0.028 |
| **N084_4** | 0.17 | 0.085 | 0.328 | 0.309 | 0.028 | 0.079 | **N062_5** | 0.531 | 0.047 | 0.172 | 0.18 | 0.037 | 0.034 |
| **N085_3** | 0.189 | 0.348 | 0.043 | 0.081 | 0.013 | 0.326 | **N064_1** | 0.191 | 0.093 | 0.241 | 0.388 | 0.046 | 0.041 |
| **N087_4** | 0.738 | 0.06 | 0.041 | 0.036 | 0.019 | 0.105 | **N069_14** | 0.623 | 0.081 | 0.079 | 0.104 | 0.057 | 0.057 |
| **N089_13** | 0.861 | 0.022 | 0.035 | 0.027 | 0.024 | 0.031 | **N070_11** | 0.531 | 0.056 | 0.102 | 0.055 | 0.223 | 0.033 |
| **N090_4** | 0.185 | 0.109 | 0.093 | 0.086 | 0.5 | 0.026 | **N017_12** | 0.733 | 0.026 | 0.045 | 0.061 | 0.021 | 0.114 |
| **N091_13** | 0.317 | 0.103 | 0.203 | 0.194 | 0.138 | 0.046 | **N018_13** | 0.651 | 0.153 | 0.019 | 0.076 | 0.025 | 0.076 |
| **N092_15** | 0.08 | 0.055 | 0.397 | 0.377 | 0.02 | 0.07 | **N022_15** | 0.237 | 0.449 | 0.034 | 0.168 | 0.008 | 0.104 |
| **N093_5** | 0.669 | 0.069 | 0.065 | 0.077 | 0.03 | 0.089 | **N023_15** | 0.511 | 0.23 | 0.027 | 0.119 | 0.015 | 0.097 |
| **N094_1** | 0.403 | 0.09 | 0.159 | 0.27 | 0.044 | 0.034 | **N024_5** | 0.769 | 0.017 | 0.023 | 0.026 | 0.043 | 0.122 |
| **N098_4** | 0.063 | 0.051 | 0.405 | 0.376 | 0.028 | 0.077 | **N045_3** | 0.672 | 0.038 | 0.027 | 0.028 | 0.055 | 0.18 |
| **N101_12** | 0.305 | 0.156 | 0.21 | 0.13 | 0.148 | 0.051 | **N046_12** | 0.706 | 0.048 | 0.018 | 0.045 | 0.025 | 0.158 |
| **N103_14** | 0.115 | 0.331 | 0.083 | 0.09 | 0.031 | 0.349 | **N048_2** | 0.679 | 0.04 | 0.037 | 0.066 | 0.05 | 0.129 |
| **N104_14** | 0.199 | 0.12 | 0.218 | 0.366 | 0.038 | 0.058 | **N50_5** | 0.724 | 0.066 | 0.022 | 0.067 | 0.029 | 0.092 |
| **N105_5** | 0.17 | 0.115 | 0.223 | 0.406 | 0.04 | 0.046 | **N25_5** | 0.864 | 0.01 | 0.045 | 0.038 | 0.043 | 0 |
| **N04_2** | 0.477 | 0.248 | 0.029 | 0.15 | 0.096 | 0 | **N26_13** | 0.658 | 0.03 | 0.063 | 0.026 | 0.22 | 0.003 |
| **N07_3** | 0.1 | 0.66 | 0.007 | 0.229 | 0.004 | 0 | **N029_11** | 0.794 | 0.036 | 0.038 | 0.083 | 0.049 | 0 |
| **N011_2** | 0.281 | 0.207 | 0.088 | 0.403 | 0.022 | 0 | **N030_12** | 0.651 | 0.035 | 0.061 | 0.048 | 0.206 | 0 |
| **N013_2** | 0.099 | 0.669 | 0.006 | 0.221 | 0.005 | 0 | **N033_14** | 0.736 | 0.013 | 0.028 | 0.017 | 0.204 | 0 |
| **N108_14** | 0.336 | 0.173 | 0.131 | 0.314 | 0.046 | 0 | **N034_4** | 0.772 | 0.011 | 0.047 | 0.014 | 0.156 | 0 |
| **N109_13** | 0.609 | 0.138 | 0.029 | 0.204 | 0.019 | 0 | **N037_3** | 0.402 | 0.017 | 0.048 | 0.019 | 0.514 | 0 |
| **N111_14** | 0.481 | 0.346 | 0.013 | 0.15 | 0.01 | 0 | **N038_2** | 0.592 | 0.009 | 0.035 | 0.015 | 0.349 | 0 |
| **N112_13** | 0.587 | 0.213 | 0.022 | 0.168 | 0.009 | 0 | **N041_1** | 0.615 | 0.034 | 0.095 | 0.105 | 0.151 | 0 |
| **N113_13** | 0.519 | 0.257 | 0.023 | 0.19 | 0.011 | 0 | **N043_13** | 0.16 | 0.035 | 0.113 | 0.054 | 0.638 | 0 |

**Supplementary Table 5.** Annealing Temperature (°C) of Microsatellite loci used

| Locus | Temperature °C |
| --- | --- |
| B124 | 55 |
| A113 | 60 |
| A24 | 55 |
| A28 | 54 |
| A88 | 55 |
| A43 | 55 |
| A007 | 56 |
| A079 | 56 |
| A107 | 55 |

**Supplementary Table 6.** Hardy-Weinberg equilibrium (HWE, right column) and linkage disequilibrium (LD, left column) tests for our large dataset (A) and our small dataset (B).

A

| Significant linkage disequilibrium | | | | | | | |  |  | Hardy-Weinberg equilibrium | | | | | | |
| --- | --- | --- | --- | --- | --- | --- | --- | --- | --- | --- | --- | --- | --- | --- | --- | --- |
| (+ = significant at level=0.008) | | | | | | | |  | Exact test using a Markov chain (for all Loci): | | | | | | | |
|  |  | |  |  |  |  |  |  |  | |  |  |  |  |  |  |
|  | | | | | | | | MKF |  | |  |  |  |  |  |  |
| Locus | | A43 | A24 | A28 | A88 | A113 | B124 |  | Locus | | #Genot | Obs.Het. | Exp.Het. | P-value | s.d. | Steps |
| A43 | | * | - | - | - | + | + |  | 1 | | 43 | 0.86047 | 0.90479 | 0.04702 | 0.00013 | 1001000 |
| A24 | | - | * | + | - | - | - |  | 2 | | 44 | 0.72727 | 0.78474 | 0.48016 | 0.00034 | 1001000 |
| A28 | | - | + | * | + | + | + |  | 3 | | 44 | 0.90909 | 0.91980 | 0.22273 | 0.00023 | 1001000 |
| A88 | | - | - | + | * | - | + |  | 4 | | 41 | 0.92683 | 0.88648 | 0.56606 | 0.00033 | 1001000 |
| A113 | | + | - | + | - | * | - |  | 5 | | 43 | 0.95349 | 0.90917 | 0.71217 | 0.00036 | 1001000 |
| B124 | | + | - | + | + | - | * |  | 6 | | 43 | 0.93023 | 0.91163 | 0.13413 | 0.00016 | 1001000 |
|  | |  |  |  |  |  |  | MKS |  | |  |  |  |  |  |  |
| Locus | | A43 | A24 | A28 | A88 | A113 | B124 |  | Locus | | #Genot | Obs.Het. | Exp.Het. | P-value | s.d. | Steps |
| A43 | | * | - | + | + | - | - |  | 1 | | 43 | 0.95349 | 0.94419 | 0.42635 | 0.00029 | 1001000 |
| A24 | | - | * | - | - | - | - |  | 2 | | 43 | 0.79070 | 0.81259 | 0.14974 | 0.00017 | 1001000 |
| A28 | | + | - | * | - | + | - |  | 3 | | 43 | 0.90698 | 0.92066 | 0.00027 | 0.00001 | 1001000 |
| A88 | | + | - | - | * | + | + |  | 4 | | 44 | 0.88636 | 0.93652 | 0.37477 | 0.00027 | 1001000 |
| A113 | | - | - | + | + | * | + |  | 5 | | 44 | 0.90909 | 0.93913 | 0.08642 | 0.00015 | 1001000 |
| B124 | | - | - | - | + | + | * |  | 6 | | 44 | 0.81818 | 0.92816 | 0.00000 | 0.00000 | 1001000 |
|  | |  |  |  |  |  |  | NHF |  | |  |  |  |  |  |  |
| Locus | | A43 | A24 | A28 | A88 | A113 | B124 |  | Locus | | #Genot | Obs.Het. | Exp.Het. | P-value | s.d. | Steps done |
| A43 | | * | - | + | + | + | + |  | 1 | | 46 | 0.80435 | 0.91687 | 0.02585 | 0.00009 | 1001000 |
| A24 | | - | * | - | - | - | - |  | 2 | | 46 | 0.84783 | 0.79025 | 0.48493 | 0.00040 | 1001000 |
| A28 | | + | - | * | + | + | + |  | 3 | | 46 | 0.91304 | 0.91639 | 0.11061 | 0.00023 | 1001000 |
| A88 | | + | - | + | * | + | + |  | 4 | | 46 | 0.93478 | 0.93120 | 0.00933 | 0.00007 | 1001000 |
| A113 | | + | - | + | + | * | + |  | 5 | | 46 | 0.84783 | 0.93096 | 0.00000 | 0.00000 | 1001000 |
| B124 | | + | - | + | + | + | * |  | 6 | | 46 | 0.89130 | 0.93550 | 0.01838 | 0.00007 | 1001000 |
|  | |  |  |  |  |  |  | NHS |  | |  |  |  |  |  |  |
| Locus | | A43 | A24 | A28 | A88 | A113 | B124 |  | Locus | | #Genot | Obs.Het. | Exp.Het. | P-value | s.d. | Steps done |
|  | |  |  |  |  |  |  |  |  | |  |  |  |  |  |  |
| A43 | | * | + | - | - | + | - |  | 1 | | 49 | 0.85714 | 0.90406 | 0.03110 | 0.00014 | 1001000 |
| A24 | | + | * | - | + | + | + |  | 2 | | 50 | 0.90000 | 0.85838 | 0.73111 | 0.00032 | 1001000 |
| A28 | | - | - | * | - | - | + |  | 3 | | 50 | 0.88000 | 0.91939 | 0.01375 | 0.00011 | 1001000 |
| A88 | | - | + | - | * | + | - |  | 4 | | 49 | 0.83673 | 0.90006 | 0.03491 | 0.00015 | 1001000 |
| A113 | | + | + | - | + | * | + |  | 5 | | 50 | 0.94000 | 0.92808 | 0.21488 | 0.00021 | 1001000 |
| B124 | | - | + | + | - | + | * |  | 6 | | 50 | 0.96000 | 0.93091 | 0.28159 | 0.00021 | 1001000 |
|  | |  |  |  |  |  |  | MF |  | |  |  |  |  |  |  |
| Locus | | A43 | A24 | A28 | A88 | A113 | B124 |  | Locus | | #Genot | Obs.Het. | Exp.Het. | P-value | s.d. | Steps done |
| A43 | | * | - | - | + | + | + |  | 1 | | 66 | 0.92424 | 0.91950 | 0.19000 | 0.00022 | 1001000 |
| A24 | | - | * | - | - | + | + |  | 2 | | 66 | 0.78788 | 0.82142 | 0.18903 | 0.00033 | 1001000 |
| A28 | | - | - | * | + | + | + |  | 3 | | 66 | 0.84848 | 0.92054 | 0.03023 | 0.00010 | 1001000 |
| A88 | | + | - | + | * | + | + |  | 4 | | 66 | 0.92424 | 0.93303 | 0.00016 | 0.00001 | 1001000 |
| A113 | | + | + | + | + | * | + |  | 5 | | 66 | 0.95455 | 0.92910 | 0.00316 | 0.00007 | 1001000 |
| B124 | | + | + | + | + | + | * |  | 6 | | 66 | 0.92424 | 0.93639 | 0.01343 | 0.00009 | 1001000 |
|  | |  |  |  |  |  |  | MS |  | |  |  |  |  |  |  |
| Locus | | A43 | A24 | A28 | A88 | A113 | B124 |  | Locus | | #Genot | Obs.Het. | Exp.Het. | P-value | s.d. | Steps done |
|  | |  |  |  |  |  |  |  |  | |  |  |  |  |  |  |
| A43 | | * | - | + | + | + | + |  | 1 | | 41 | 0.90244 | 0.92141 | 0.00254 | 0.00003 | 1001000 |
| A24 | | - | * | + | - | + | + |  | 2 | | 44 | 0.75000 | 0.79101 | 0.34690 | 0.00029 | 1001000 |
| A28 | | + | + | * | + | + | + |  | 3 | | 44 | 0.84091 | 0.90361 | 0.00267 | 0.00004 | 1001000 |
| A88 | | + | - | + | * | + | + |  | 4 | | 43 | 0.88372 | 0.93598 | 0.06522 | 0.00022 | 1001000 |
| A113 | | + | + | + | + | * | + |  | 5 | | 44 | 0.90909 | 0.92268 | 0.38024 | 0.00027 | 1001000 |
| B124 | | + | + | + | + | + | * |  | 6 | | 44 | 0.93182 | 0.93757 | 0.17484 | 0.00019 | 1001000 |

B. Significant linkage disequilibrium (+ = significant at level at 0.05) and Hardy-Weinberg equilibrium exact test using a Markov chain (right panel) for the small dataset.

|  |  |  |  |  |  |  |  |  |  | MF |  |  |  |  |  |  |
| --- | --- | --- | --- | --- | --- | --- | --- | --- | --- | --- | --- | --- | --- | --- | --- | --- |
| Locus | A43 | A24 | A28 | A88 | A113 | B124 | A107 | A079 | A007 |  | Locus | #Genot | Obs.Het. | Exp.Het. | P-value | s.d. |
| A43 | * | - | - | - | - | - | - | - | - |  | A43 | 10 | 0.9 | 0.96316 | 0.38965 | 0.00021 |
| A24 | - | * | - | - | - | - | - | - | - |  | A24 | 10 | 0.6 | 0.77368 | 0.18086 | 0.00035 |
| A28 | - | - | * | - | - | - | + | + | - |  | A28 | 10 | 0.8 | 0.93684 | 0.2123 | 0.00046 |
| A88 | - | - | - | * | + | - | - | - | - |  | A88 | 10 | 0.9 | 0.95263 | 0.54825 | 0.00023 |
| A113 | - | - | - | + | * | - | - | - | - |  | A113 | 10 | 0.9 | 0.88947 | 0.95247 | 0.00021 |
| B124 | - | - | - | - | - | * | - | - | - |  | B124 | 10 | 0.9 | 0.94211 | 0.59854 | 0.00042 |
| A107 | - | - | + | - | - | - | * | + | + |  | A107 | 8 | 0.125 | 0.875 | 0 | 0 |
| A079 | - | - | + | - | - | - | + | * | - |  | A079 | 10 | 0.7 | 0.9 | 0.19532 | 0.00049 |
| A007 | - | - | - | - | - | - | + | - | * |  | A007 | 10 | 0.6 | 0.86316 | 0.08606 | 0.00029 |
|  |  |  |  |  |  |  |  |  |  | MS |  |  |  |  |  |  |
| Locus | A43 | A24 | A28 | A88 | A113 | B124 | A107 | A079 | A007 |  | Locus | #Genot | Obs.Het. | Exp.Het. | P-value | s.d. |
| A43 | * | - | - | - | - | - | - | - | - |  | A43 | 9 | 0.88889 | 0.88235 | 0.94788 | 0.00027 |
| A24 | - | * | - | - | - | - | - | - | - |  | A24 | 9 | 0.66667 | 0.68627 | 0.41369 | 0.00044 |
| A28 | - | - | * | - | - | - | + | + | - |  | A28 | 9 | 0.77778 | 0.88889 | 0.53739 | 0.00046 |
| A88 | - | - | - | * | + | - | - | - | - |  | A88 | 9 | 0.88889 | 0.90196 | 0.14734 | 0.00029 |
| A113 | - | - | - | + | * | - | - | - | - |  | A113 | 9 | 0.77778 | 0.91503 | 0.12029 | 0.00037 |
| B124 | - | - | - | - | - | * | - | - | - |  | B124 | 9 | 0.77778 | 0.94771 | 0.13218 | 0.00033 |
| A107 | - | - | + | - | - | - | * | + | + |  | A107 | 9 | 0 | 0.88889 | 0 | 0 |
| A079 | - | - | + | - | - | - | + | * | - |  | A079 | 8 | 0.5 | 0.9 | 0.0028 | 0.00004 |
| A007 | - | - | - | - | - | - | + | - | * |  | A007 | 9 | 0.33333 | 0.90196 | 0 | 0 |
|  |  |  |  |  |  |  |  |  |  | MKF |  |  |  |  |  |  |
| Locus | A43 | A24 | A28 | A88 | A113 | B124 | A107 | A079 | A007 |  | Locus | #Genot | Obs.Het. | Exp.Het. | P-value | s.d. |
| A43 | * | - | - | - | - | - | - | + | - |  | A43 | 9 | 1 | 0.92157 | 1 | 0 |
| A24 | - | * | - | - | - | - | - | - | - |  | A24 | 9 | 0.55556 | 0.64706 | 0.84053 | 0.00037 |
| A28 | - | - | * | - | - | - | + | - | + |  | A28 | 9 | 0.44444 | 0.9281 | 0 | 0 |
| A88 | - | - | - | * | - | - | + | - | - |  | A88 | 9 | 0.77778 | 0.9085 | 0.10803 | 0.00029 |
| A113 | - | - | - | - | * | - | - | - | - |  | A113 | 9 | 0.88889 | 0.9085 | 0.81441 | 0.00032 |
| B124 | - | - | - | - | - | * | - | + | - |  | B124 | 9 | 0.88889 | 0.9281 | 0.19771 | 0.00029 |
| A107 | - | - | + | + | - | - | * | - | + |  | A107 | 5 | 0.2 | 0.73333 | 0.01607 | 0.00012 |
| A079 | + | - | - | - | - | + | - | * | - |  | A079 | 9 | 0.55556 | 0.77124 | 0.10726 | 0.00025 |
| A007 | - | - | + | - | - | - | + | - | * |  | A007 | 9 | 0.33333 | 0.90196 | 0.00013 | 0.00001 |
|  |  |  |  |  |  |  |  |  |  | MKS |  |  |  |  |  |  |
| Locus | A43 | A24 | A28 | A88 | A113 | B124 | A107 | A079 | A007 |  | Locus | #Genot | Obs.Het. | Exp.Het. | P-value | s.d. |
| A43 | * | - | - | - | - | + | - | + | - |  | A43 | 9 | 0.66667 | 0.93464 | 0.04544 | 0.00017 |
| A24 | - | * | - | - | - | - | - | - | - |  | A24 | 9 | 0.88889 | 0.77124 | 0.76742 | 0.00041 |
| A28 | - | - | * | - | - | - | - | - | - |  | A28 | 9 | 0.88889 | 0.89542 | 0.46505 | 0.00034 |
| A88 | - | - | - | * | - | + | - | - | - |  | A88 | 9 | 0.66667 | 0.91503 | 0.00783 | 0.00006 |
| A113 | - | - | - | - | * | - | - | - | - |  | A113 | 9 | 0.66667 | 0.86275 | 0.13893 | 0.00033 |
| B124 | + | - | - | + | - | * | - | - | - |  | B124 | 9 | 0.66667 | 0.96078 | 0.00266 | 0.00004 |
| A107 | - | - | - | - | - | - | * | - | - |  | A107 | 7 | 0.28571 | 0.85714 | 0.00044 | 0.00002 |
| A079 | + | - | - | - | - | - | - | * | - |  | A079 | 9 | 0.77778 | 0.86928 | 0.26394 | 0.00029 |
| A007 | - | - | - | - | - | - | - | - | * |  | A007 | 9 | 0.55556 | 0.8366 | 0.02857 | 0.00016 |
|  |  |  |  |  |  |  |  |  |  | NHF |  |  |  |  |  |  |
| Locus | A43 | A24 | A28 | A88 | A113 | B124 | A107 | A079 | A007 |  | Locus | #Genot | Obs.Het. | Exp.Het. | P-value | s.d. |
| A43 | * | - | - | - | - | + | - | + | - |  | A43 | 9 | 0.77778 | 0.91503 | 0.06538 | 0.00024 |
| A24 | - | * | - | - | - | - | - | - | - |  | A24 | 9 | 0.77778 | 0.82353 | 0.60124 | 0.00045 |
| A28 | - | - | * | - | - | - | - | - | - |  | A28 | 9 | 0.77778 | 0.91503 | 0.32426 | 0.00035 |
| A88 | - | - | - | * | - | + | - | - | - |  | A88 | 9 | 0.66667 | 0.91503 | 0.01572 | 0.00014 |
| A113 | - | - | - | - | * | - | - | - | - |  | A113 | 9 | 0.66667 | 0.88235 | 0.27929 | 0.00038 |
| B124 | + | - | - | + | - | * | - | - | - |  | B124 | 9 | 0.88889 | 0.94118 | 0.55763 | 0.00032 |
| A107 | - | - | - | - | - | - | * | - | - |  | A107 | 6 | 0 | 0.90909 | 0 | 0 |
| A079 | + | - | - | - | - | - | - | * | - |  | A079 | 9 | 0.66667 | 0.89542 | 0.17881 | 0.0003 |
| A007 | - | - | - | - | - | - | - | - | * |  | A007 | 9 | 0.55556 | 0.84314 | 0.09143 | 0.00026 |
|  |  |  |  |  |  |  |  |  |  | NHS |  |  |  |  |  |  |
| Locus | A43 | A24 | A28 | A88 | A113 | B124 | A107 | A079 | A007 |  | Locus | #Genot | Obs.Het. | Exp.Het. | P-value | s.d. |
| A43 | * | - | - | - | - | + | + | - | - |  | A43 | 10 | 0.8 | 0.92632 | 0.07467 | 0.00016 |
| A24 | - | * | - | - | - | - | - | + | - |  | A24 | 10 | 0.7 | 0.80526 | 0.35427 | 0.00046 |
| A28 | - | - | * | - | - | - | + | - | - |  | A28 | 10 | 0.7 | 0.89474 | 0.2377 | 0.00021 |
| A88 | - | - | - | * | - | - | - | - | - |  | A88 | 10 | 0.9 | 0.91579 | 0.09132 | 0.00019 |
| A113 | - | - | - | - | * | - | - | - | - |  | A113 | 10 | 1 | 0.92632 | 1 | 0 |
| B124 | + | - | - | - | - | * | + | - | - |  | B124 | 10 | 0.8 | 0.95789 | 0.08767 | 0.00023 |
| A107 | + | - | + | - | - | + | * | - | + |  | A107 | 8 | 0.375 | 0.85 | 0.00361 | 0.00005 |
| A079 | - | + | - | - | - | - | - | * | - |  | A079 | 10 | 0.8 | 0.90526 | 0.50041 | 0.00042 |
| A007 | - | - | - | - | - | - | + | - | * |  | A007 | 10 | 0.6 | 0.8 | 0.0732 | 0.00022 |

**Supplementary Table 7.** Number of individuals migrants out of 56 samples detected by Geneclass 2,

with a probability below 0.01 to belong to their home population.

**Supplementary Table 8.** Bottleneck test for all populations, using all loci and the large dataset. Acronyms used: IAM, Infinite Allele Model; SMM, Stepwise Mutation Model and TPM, Two-phase model.

| **Population** | n | Sign test | | | Allele frequency distribution |
| --- | --- | --- | --- | --- | --- |
|  |  | IAM | SMM | TPM | (L-shaped or Mode-shift ) |
| **Mount Kenya Savanna** | 44 | 0,05 | 0,54 | 0,53 | L-shaped |
| **Mount Kenya Forest** | 44 | 0,05 | 0,04 | 0,51 | L-shaped |
| **Nyambene Hills Savanna** | 50 | 0,05 | 0,48 | 0,04 | L-shaped |
| **Nyambene Hills Forest** | 46 | 0,05 | 0,18 | 0,57 | L-shaped |
| **Mau Savanna** | 44 | 0,24 | 0,52 | 0,47 | L-shaped |
| **Mau Forest** | 66 | 0,05 | 0,17 | 0,22 | L-shaped |
| **Mode-shift = evidence of bottleneck** | | | | | |

**Supporting Information : Appendix S2**

Additional data providing details on genetic analysis of *A. mellifera* populations.

Figure 1

Results of Bayesian population structure analysis as implemented in the program STRUCTURE for the nine microsatellite data obtained for the large dataset. The model employed by STRUCTURE assumes that there are “K” ancestral populations at HWE and in LD, however, the assumption of HWE and LD are not fulfilled by this large dataset. Therefore, we used the method proposed by Ostrowski *et al*. (2006) to relax these assumptions, which imply statistical independence between the two alleles present at each locus. Briefly, this method considers each individual as being haploid, which means that each of our diploid individual will now be considered as two individuals, each carrying one of the two alleles. Thus, we renamed half of our individuals and produced a new dataset consisting of 588 samples, which were used for the STRUCTUE analyses below. Each vertical line represents the proportions of individual multilocus genotypes assigned to each of the K clusters estimated by the program. Figures show the resulting number of clusters that best fit the data for each of the two assumptions used, determined by the method of Evano et al. (2005). A. Not using the LOCPRIOR option, Ln’(K = 4): -2693.4. B. Using as priors each of the six localities (MKF, MKS, MF, MS, NHF, NHS), Ln’(K = 6): -2855.525. Acronyms used: Mount Kenya Forest (MKF), Mount Kenya Savanna (MKS), Mau Forest (MF), Mau Savanna (MS), Nyambene Hills Forest (NHF), Nyambene Hills Savanna (NHS).

A.


B.

Table 1. List of individuals used for morphometric analyses.

| Site | Colony | Individual | Putative subspecies |
| --- | --- | --- | --- |
| MF | N72 | 4 | monticola |
| MF | N72 | 5 | monticola |
| MF | N72 | 6 | monticola |
| MF | N78 | 4 | monticola |
| MF | N78 | 5 | monticola |
| MF | N78 | 6 | monticola |
| MF | N81 | 4 | monticola |
| MF | N81 | 5 | monticola |
| MF | N81 | 11 | monticola |
| MF | N85 | 6 | monticola |
| MF | N85 | 11 | monticola |
| MF | N89 | 5 | monticola |
| MF | N89 | 6 | monticola |
| MF | N89 | 7 | monticola |
| MF | N89 | 8 | monticola |
| MF | N89 | 9 | monticola |
| MF | N89 | 10 | monticola |
| MKF | N108 | 1 | monticola |
| MKF | N108 | 3 | monticola |
| MKF | N108 | 4 | monticola |
| MKF | N108 | 5 | monticola |
| MKF | N108 | 7 | monticola |
| MKF | N108 | 9 | monticola |
| MKF | N109 | 1 | monticola |
| MKF | N109 | 2 | monticola |
| MKF | N109 | 3 | monticola |
| MKF | N109 | 4 | monticola |
| MKF | N109 | 5 | monticola |
| MKF | N109 | 6 | monticola |
| MKF | N109 | 11 | monticola |
| MKF | N111 | 1 | monticola |
| MKF | N111 | 2 | monticola |
| MKF | N111 | 4 | monticola |
| MKF | N111 | 5 | monticola |
| MKF | N111 | 11 | monticola |
| MKF | N112 | 1 | monticola |
| MKF | N112 | 3 | monticola |
| MKF | N112 | 4 | monticola |
| MKF | N112 | 5 | monticola |
| MKF | N112 | 11 | monticola |
| MKF | N113 | 2 | monticola |
| MKF | N113 | 3 | monticola |
| MKF | N113 | 4 | monticola |
| MKF | N113 | 5 | monticola |
| MKF | N113 | 11 | monticola |
| MKF | N113 | 14 | monticola |
| MKS | N55 | 1 | scutellata |
| MKS | N55 | 2 | scutellata |
| MKS | N55 | 3 | scutellata |
| MKS | N55 | 4 | scutellata |
| MKS | N55 | 5 | scutellata |
| MKS | N55 | 6 | scutellata |
| MKS | N55 | 7 | scutellata |
| MKS | N55 | 8 | scutellata |
| MKS | N55 | 9 | scutellata |
| MKS | N56 | 2 | scutellata |
| MKS | N56 | 3 | scutellata |
| MKS | N56 | 4 | scutellata |
| MKS | N56 | 5 | scutellata |
| MKS | N56 | 7 | scutellata |
| MKS | N56 | 8 | scutellata |
| MKS | N56 | 9 | scutellata |
| MKS | N56 | 10 | scutellata |
| MKS | N56 | 11 | scutellata |
| MKS | N60 | 1 | scutellata |
| MKS | N60 | 2 | scutellata |
| MKS | N60 | 3 | scutellata |
| MKS | N60 | 4 | scutellata |
| MKS | N60 | 5 | scutellata |
| MKS | N60 | 6 | scutellata |
| MKS | N60 | 7 | scutellata |
| MKS | N60 | 8 | scutellata |
| MKS | N60 | 9 | scutellata |
| MKS | N60 | 10 | scutellata |
| MKS | N69 | 1 | scutellata |
| MKS | N69 | 2 | scutellata |
| MKS | N69 | 3 | scutellata |
| MKS | N69 | 4 | scutellata |
| MKS | N69 | 5 | scutellata |
| MKS | N69 | 7 | scutellata |
| MKS | N69 | 8 | scutellata |
| MKS | N69 | 9 | scutellata |
| MKS | N69 | 10 | scutellata |
| MKS | N70 | 1 | scutellata |
| MKS | N70 | 2 | scutellata |
| MKS | N70 | 3 | scutellata |
| MKS | N70 | 4 | scutellata |
| MKS | N70 | 5 | scutellata |
| MKS | N70 | 6 | scutellata |
| MKS | N70 | 7 | scutellata |
| MKS | N70 | 8 | scutellata |
| MKS | N70 | 9 | scutellata |
| MKS | N70 | 10 | scutellata |
| MS | N101 | 1 | scutellata |
| MS | N101 | 2 | scutellata |
| MS | N101 | 4 | scutellata |
| MS | N101 | 5 | scutellata |
| MS | N101 | 6 | scutellata |
| MS | N101 | 8 | scutellata |
| MS | N101 | 9 | scutellata |
| MS | N101 | 11 | scutellata |
| MS | N103 | 1 | scutellata |
| MS | N103 | 2 | scutellata |
| MS | N103 | 3 | scutellata |
| MS | N103 | 4 | scutellata |
| MS | N103 | 5 | scutellata |
| MS | N103 | 6 | scutellata |
| MS | N103 | 7 | scutellata |
| MS | N103 | 8 | scutellata |
| MS | N103 | 9 | scutellata |
| MS | N103 | 10 | scutellata |
| MS | N104 | 1 | scutellata |
| MS | N104 | 2 | scutellata |
| MS | N104 | 3 | scutellata |
| MS | N104 | 4 | scutellata |
| MS | N104 | 6 | scutellata |
| MS | N104 | 7 | scutellata |
| MS | N104 | 8 | scutellata |
| MS | N104 | 9 | scutellata |
| MS | N104 | 10 | scutellata |
| MS | N91 | 1 | scutellata |
| MS | N91 | 2 | scutellata |
| MS | N91 | 3 | scutellata |
| MS | N91 | 4 | scutellata |
| MS | N91 | 5 | scutellata |
| MS | N91 | 7 | scutellata |
| MS | N91 | 8 | scutellata |
| MS | N91 | 9 | scutellata |
| MS | N91 | 10 | scutellata |
| MS | N92 | 1 | scutellata |
| MS | N92 | 2 | scutellata |
| MS | N92 | 3 | scutellata |
| MS | N92 | 4 | scutellata |
| MS | N92 | 5 | scutellata |
| MS | N92 | 6 | scutellata |
| MS | N92 | 8 | scutellata |
| MS | N92 | 9 | scutellata |
| MS | N92 | 10 | scutellata |
| NHF | N17 | 1 | monticola |
| NHF | N17 | 2 | monticola |
| NHF | N17 | 3 | monticola |
| NHF | N17 | 5 | monticola |
| NHF | N17 | 6 | monticola |
| NHF | N18 | 1 | monticola |
| NHF | N18 | 2 | monticola |
| NHF | N18 | 3 | monticola |
| NHF | N18 | 4 | monticola |
| NHF | N18 | 5 | monticola |
| NHF | N18 | 6 | monticola |
| NHF | N18 | 7 | monticola |
| NHF | N22 | 2 | monticola |
| NHF | N22 | 3 | monticola |
| NHF | N22 | 4 | monticola |
| NHF | N22 | 5 | monticola |
| NHF | N22 | 6 | monticola |
| NHF | N22 | 7 | monticola |
| NHF | N22 | 11 | monticola |
| NHF | N23 | 1 | monticola |
| NHF | N23 | 2 | monticola |
| NHF | N23 | 3 | monticola |
| NHF | N23 | 4 | monticola |
| NHF | N23 | 5 | monticola |
| NHF | N23 | 6 | monticola |
| NHF | N23 | 7 | monticola |
| NHF | N23 | 9 | monticola |
| NHF | N46 | 1 | monticola |
| NHF | N46 | 2 | monticola |
| NHF | N46 | 3 | monticola |
| NHF | N46 | 4 | monticola |
| NHF | N46 | 5 | monticola |
| NHF | N46 | 7 | monticola |
| NHF | N46 | 8 | monticola |
| NHS | N26 | 1 | scutellata |
| NHS | N26 | 2 | scutellata |
| NHS | N26 | 3 | scutellata |
| NHS | N26 | 4 | scutellata |
| NHS | N26 | 5 | scutellata |
| NHS | N26 | 6 | scutellata |
| NHS | N26 | 7 | scutellata |
| NHS | N26 | 8 | scutellata |
| NHS | N26 | 9 | scutellata |
| NHS | N26 | 10 | scutellata |
| NHS | N29 | 1 | scutellata |
| NHS | N29 | 2 | scutellata |
| NHS | N29 | 3 | scutellata |
| NHS | N29 | 4 | scutellata |
| NHS | N29 | 5 | scutellata |
| NHS | N29 | 6 | scutellata |
| NHS | N29 | 7 | scutellata |
| NHS | N29 | 8 | scutellata |
| NHS | N29 | 9 | scutellata |
| NHS | N29 | 10 | scutellata |
| NHS | N30 | 1 | scutellata |
| NHS | N30 | 2 | scutellata |
| NHS | N30 | 3 | scutellata |
| NHS | N30 | 4 | scutellata |
| NHS | N30 | 5 | scutellata |
| NHS | N30 | 7 | scutellata |
| NHS | N30 | 9 | scutellata |
| NHS | N30 | 10 | scutellata |
| NHS | N30 | 11 | scutellata |
| NHS | N33 | 1 | scutellata |
| NHS | N33 | 2 | scutellata |
| NHS | N33 | 3 | scutellata |
| NHS | N33 | 4 | scutellata |
| NHS | N33 | 5 | scutellata |
| NHS | N33 | 6 | scutellata |
| NHS | N33 | 7 | scutellata |
| NHS | N33 | 8 | scutellata |
| NHS | N33 | 9 | scutellata |
| NHS | N33 | 10 | scutellata |
| NHS | N43 | 1 | scutellata |
| NHS | N43 | 3 | scutellata |
| NHS | N43 | 4 | scutellata |
| NHS | N43 | 5 | scutellata |
| NHS | N43 | 6 | scutellata |
| NHS | N43 | 7 | scutellata |
| NHS | N43 | 8 | scutellata |
| NHS | N43 | 9 | scutellata |
| NHS | N43 | 10 | scutellata |
| MF | N072 | 13 | monticola |
| MF | N081 | 1 | monticola |
| MF | N081 | 2 | monticola |
| MF | N081 | 3 | monticola |
| MF | N081 | 12 | monticola |
| MF | N085 | 1 | monticola |
| MF | N085 | 2 | monticola |
| MF | N085 | 3 | monticola |
| MF | N085 | 4 | monticola |
| MF | N085 | 12 | monticola |
| MKF | N108 | 11 | monticola |
| MKF | N108 | 14 | monticola |
| MKF | N108 | 15 | monticola |
| MKF | N112 | 12 | monticola |
| MKF | N113 | 13 | monticola |
| MS | N092 | 7 | scutellata |
| MS | N101 | 10 | scutellata |
| MS | N104 | 5 | scutellata |
| NHF | N017 | 4 | monticola |
| NHS | N030 | 6 | scutellata |
| MF | N072 | 1 | monticola |
| MF | N072 | 2 | monticola |
| MF | N072 | 3 | monticola |
| MF | N072 | 11 | monticola |
| MF | N072 | 14 | monticola |
| MF | N072 | 15 | monticola |
| MF | N078 | 1 | monticola |
| MF | N078 | 2 | monticola |
| MF | N078 | 3 | monticola |
| MF | N078 | 11 | monticola |
| MF | N078 | 12 | monticola |
| MF | N078 | 14 | monticola |
| MF | N078 | 15 | monticola |
| MF | N081 | 13 | monticola |
| MF | N081 | 14 | monticola |
| MF | N081 | 15 | monticola |
| MF | N085 | 13 | monticola |
| MF | N085 | 14 | monticola |
| MF | N085 | 15 | monticola |
| MF | N089 | 1 | monticola |
| MF | N089 | 2 | monticola |
| MF | N089 | 3 | monticola |
| MF | N089 | 4 | monticola |
| MKF | N108 | 12 | monticola |
| MKF | N109 | 13 | monticola |
| MKF | N109 | 14 | monticola |
| MKF | N109 | 15 | monticola |
| MKF | N111 | 3 | monticola |
| MKF | N111 | 12 | monticola |
| MKF | N111 | 13 | monticola |
| MKF | N111 | 14 | monticola |
| MKF | N111 | 15 | monticola |
| MKF | N112 | 2 | monticola |
| MKF | N112 | 13 | monticola |
| MKF | N112 | 14 | monticola |
| MKF | N112 | 15 | monticola |
| MKF | N113 | 1 | monticola |
| MKF | N113 | 12 | monticola |
| MKF | N113 | 15 | monticola |
| MKS | N055 | 10 | scutellata |
| MKS | N056 | 1 | scutellata |
| MKS | N069 | 11 | scutellata |
| MS | N091 | 11 | scutellata |
| MS | N101 | 12 | scutellata |
| NHF | N017 | 11 | monticola |
| NHF | N017 | 13 | monticola |
| NHF | N017 | 14 | monticola |
| NHF | N017 | 15 | monticola |
| NHF | N018 | 12 | monticola |
| NHF | N018 | 14 | monticola |
| NHF | N018 | 15 | monticola |
| NHF | N022 | 13 | monticola |
| NHF | N022 | 14 | monticola |
| NHF | N022 | 15 | monticola |
| NHF | N023 | 11 | monticola |
| NHF | N023 | 13 | monticola |
| NHF | N046 | 6 | monticola |
| NHF | N046 | 13 | monticola |
| NHF | N046 | 15 | monticola |
| NHS | N043 | 11 | scutellata |

Table 2. List of individuals used for mitochondrial analyses.

| Site | Colony | Individual | Putative subspecies |
| --- | --- | --- | --- |
| MF | N072 | 12 | monticola |
| MF | N073 | 4 | monticola |
| MF | N078 | 13 | monticola |
| MF | N078 | 1 | monticola |
| MF | N081 | 1 | monticola |
| MF | N084 | 1 | monticola |
| MF | N085 | 13 | monticola |
| MF | N087 | 1 | monticola |
| MF | N089 | 1 | monticola |
| MF | N090 | 1 | monticola |
| MKF | N004 | 1 | monticola |
| MKF | N007 | 1 | monticola |
| MKF | N011 | 1 | monticola |
| MKF | N013 | 1 | monticola |
| MKF | N108 | 12 | monticola |
| MKF | N109 | 13 | monticola |
| MKF | N111 | 3 | monticola |
| MKF | N112 | 2 | monticola |
| MKF | N113 | 1 | monticola |
| MKS | N056 | 1 | scutellata |
| MKS | N060 | 11 | scutellata |
| MKS | N069 | 11 | scutellata |
| MKS | N070 | 11 | scutellata |
| MS | N091 | 11 | scutellata |
| MS | N092 | 12 | scutellata |
| MS | N101 | 12 | scutellata |
| MS | N103 | 14 | scutellata |
| NHF | N017 | 11 | monticola |
| NHF | N018 | 12 | monticola |
| NHF | N022 | 1 | monticola |
| NHF | N023 | 11 | monticola |
| NHF | N024 | 1 | monticola |
| NHF | N045 | 1 | monticola |
| NHF | N046 | 6 | monticola |
| NHF | N048 | 1 | monticola |
| NHF | N050 | 2 | monticola |
| NHS | N026 | 11 | scutellata |
| NHS | N029 | 11 | scutellata |
| NHS | N030 | 12 | scutellata |
| NHS | N033 | 11 | scutellata |
| NHS | N043 | 11 | scutellata |

Table 3

List of individuals of the small dataset used for microsatellite analyses

| Site | Colony | Individual | Putative subspecies | Site | Colony | Individual | Putative subspecies |
| --- | --- | --- | --- | --- | --- | --- | --- |
| MF | N071 | 4 | monticola | MKS | N014 | 1 | scutellata |
| MF | N072 | 1 | monticola | MKS | N016 | 5 | scutellata |
| MF | N073 | 1 | monticola | MKS | N055 | 15 | scutellata |
| MF | N078 | 2 | monticola | MKS | N056 | 13 | scutellata |
| MF | N081 | 2 | monticola | MKS | N060 | 13 | scutellata |
| MF | N084 | 4 | monticola | MKS | N062 | 5 | scutellata |
| MF | N085 | 3 | monticola | MKS | N064 | 1 | scutellata |
| MF | N087 | 4 | monticola | MKS | N069 | 14 | scutellata |
| MF | N089 | 13 | monticola | MKS | N070 | 11 | scutellata |
| MF | N090 | 4 | monticola | NHF | N017 | 12 | monticola |
| MS | N091 | 13 | scutellata | NHF | N018 | 13 | monticola |
| MS | N092 | 15 | scutellata | NHF | N022 | 15 | monticola |
| MS | N093 | 5 | scutellata | NHF | N023 | 15 | monticola |
| MS | N094 | 1 | scutellata | NHF | N024 | 5 | monticola |
| MS | N098 | 4 | scutellata | NHF | N045 | 3 | monticola |
| MS | N101 | 12 | scutellata | NHF | N046 | 12 | monticola |
| MS | N103 | 14 | scutellata | NHF | N048 | 2 | monticola |
| MS | N104 | 14 | scutellata | NHF | N50 | 5 | monticola |
| MS | N105 | 5 | scutellata | NHS | N25 | 5 | scutellata |
| MKF | N04 | 2 | monticola | NHS | N26 | 13 | scutellata |
| MKF | N07 | 3 | monticola | NHS | N029 | 11 | scutellata |
| MKF | N011 | 2 | monticola | NHS | N030 | 12 | scutellata |
| MKF | N013 | 2 | monticola | NHS | N033 | 14 | scutellata |
| MKF | N108 | 14 | monticola | NHS | N034 | 4 | scutellata |
| MKF | N109 | 13 | monticola | NHS | N037 | 3 | scutellata |
| MKF | N111 | 14 | monticola | NHS | N038 | 2 | scutellata |
| MKF | N112 | 13 | monticola | NHS | N041 | 1 | scutellata |
| MKF | N113 | 13 | monticola | NHS | N043 | 13 | scutellata |

Table 4. List of individuals from the larger dataset, used for microsatellite analyses.

| Site | Colony | Individual | Putative subspecies |
| --- | --- | --- | --- |
| MF | N071 | 1 | monticola |
| MF | N071 | 2 | monticola |
| MF | N071 | 3 | monticola |
| MF | N071 | 4 | monticola |
| MF | N071 | 5 | monticola |
| MF | N072 | 13 | monticola |
| MF | N073 | 1 | monticola |
| MF | N073 | 2 | monticola |
| MF | N073 | 3 | monticola |
| MF | N073 | 5 | monticola |
| MF | N081 | 8 | monticola |
| MF | N081 | 1 | monticola |
| MF | N081 | 2 | monticola |
| MF | N081 | 3 | monticola |
| MF | N081 | 12 | monticola |
| MF | N084 | 2 | monticola |
| MF | N084 | 3 | monticola |
| MF | N084 | 4 | monticola |
| MF | N085 | 5 | monticola |
| MF | N085 | 1 | monticola |
| MF | N085 | 2 | monticola |
| MF | N085 | 3 | monticola |
| MF | N085 | 4 | monticola |
| MF | N085 | 12 | monticola |
| MF | N087 | 2 | monticola |
| MF | N087 | 3 | monticola |
| MF | N087 | 4 | monticola |
| MF | N089 | 12 | monticola |
| MF | N089 | 14 | monticola |
| MF | N090 | 2 | monticola |
| MF | N090 | 3 | monticola |
| MF | N090 | 4 | monticola |
| MKF | N004 | 2 | monticola |
| MKF | N004 | 3 | monticola |
| MKF | N004 | 4 | monticola |
| MKF | N007 | 2 | monticola |
| MKF | N007 | 3 | monticola |
| MKF | N007 | 4 | monticola |
| MKF | N007 | 5 | monticola |
| MKF | N011 | 2 | monticola |
| MKF | N011 | 3 | monticola |
| MKF | N011 | 4 | monticola |
| MKF | N011 | 5 | monticola |
| MKF | N013 | 2 | monticola |
| MKF | N013 | 3 | monticola |
| MKF | N013 | 4 | monticola |
| MKF | N013 | 5 | monticola |
| MKF | N108 | 13 | monticola |
| MKF | N108 | 11 | monticola |
| MKF | N108 | 14 | monticola |
| MKF | N108 | 15 | monticola |
| MKF | N109 | 7 | monticola |
| MKF | N109 | 12 | monticola |
| MKF | N112 | 12 | monticola |
| MKF | N113 | 13 | monticola |
| MKS | N014 | 1 | scutellata |
| MKS | N014 | 2 | scutellata |
| MKS | N014 | 3 | scutellata |
| MKS | N014 | 4 | scutellata |
| MKS | N016 | 1 | scutellata |
| MKS | N016 | 2 | scutellata |
| MKS | N016 | 3 | scutellata |
| MKS | N016 | 4 | scutellata |
| MKS | N016 | 5 | scutellata |
| MKS | N055 | 15 | scutellata |
| MKS | N056 | 12 | scutellata |
| MKS | N056 | 14 | scutellata |
| MKS | N060 | 13 | scutellata |
| MKS | N062 | 1 | scutellata |
| MKS | N062 | 2 | scutellata |
| MKS | N062 | 3 | scutellata |
| MKS | N062 | 4 | scutellata |
| MKS | N062 | 5 | scutellata |
| MKS | N064 | 1 | scutellata |
| MKS | N064 | 2 | scutellata |
| MKS | N064 | 3 | scutellata |
| MKS | N064 | 4 | scutellata |
| MKS | N064 | 5 | scutellata |
| MKS | N069 | 12 | scutellata |
| MS | N092 | 7 | scutellata |
| MS | N093 | 1 | scutellata |
| MS | N093 | 2 | scutellata |
| MS | N093 | 3 | scutellata |
| MS | N093 | 4 | scutellata |
| MS | N093 | 5 | scutellata |
| MS | N094 | 1 | scutellata |
| MS | N094 | 2 | scutellata |
| MS | N094 | 3 | scutellata |
| MS | N094 | 4 | scutellata |
| MS | N094 | 5 | scutellata |
| MS | N098 | 1 | scutellata |
| MS | N098 | 2 | scutellata |
| MS | N098 | 3 | scutellata |
| MS | N098 | 4 | scutellata |
| MS | N098 | 5 | scutellata |
| MS | N101 | 10 | scutellata |
| MS | N103 | 12 | scutellata |
| MS | N103 | 13 | scutellata |
| MS | N104 | 12 | scutellata |
| MS | N104 | 13 | scutellata |
| MS | N104 | 14 | scutellata |
| MS | N104 | 15 | scutellata |
| MS | N104 | 5 | scutellata |
| MS | N105 | 1 | scutellata |
| MS | N105 | 2 | scutellata |
| MS | N105 | 3 | scutellata |
| MS | N105 | 4 | scutellata |
| MS | N105 | 5 | scutellata |
| NHF | N017 | 4 | monticola |
| NHF | N018 | 10 | monticola |
| NHF | N024 | 2 | monticola |
| NHF | N024 | 3 | monticola |
| NHF | N024 | 4 | monticola |
| NHF | N024 | 5 | monticola |
| NHF | N045 | 2 | monticola |
| NHF | N045 | 3 | monticola |
| NHF | N045 | 4 | monticola |
| NHF | N045 | 5 | monticola |
| NHF | N048 | 2 | monticola |
| NHF | N048 | 3 | monticola |
| NHF | N048 | 4 | monticola |
| NHF | N048 | 5 | monticola |
| NHF | N050 | 1 | monticola |
| NHF | N050 | 3 | monticola |
| NHF | N050 | 4 | monticola |
| NHF | N050 | 5 | monticola |
| NHS | N025 | 1 | scutellata |
| NHS | N025 | 2 | scutellata |
| NHS | N025 | 3 | scutellata |
| NHS | N025 | 4 | scutellata |
| NHS | N025 | 5 | scutellata |
| NHS | N026 | 12 | scutellata |
| NHS | N030 | 14 | scutellata |
| NHS | N030 | 15 | scutellata |
| NHS | N030 | 6 | scutellata |
| NHS | N034 | 1 | scutellata |
| NHS | N034 | 2 | scutellata |
| NHS | N034 | 3 | scutellata |
| NHS | N034 | 4 | scutellata |
| NHS | N034 | 5 | scutellata |
| NHS | N037 | 1 | scutellata |
| NHS | N037 | 2 | scutellata |
| NHS | N037 | 3 | scutellata |
| NHS | N037 | 4 | scutellata |
| NHS | N037 | 5 | scutellata |
| NHS | N038 | 1 | scutellata |
| NHS | N038 | 2 | scutellata |
| NHS | N038 | 3 | scutellata |
| NHS | N038 | 4 | scutellata |
| NHS | N038 | 5 | scutellata |
| NHS | N041 | 1 | scutellata |
| NHS | N041 | 2 | scutellata |
| NHS | N041 | 3 | scutellata |
| NHS | N041 | 4 | scutellata |
| NHS | N041 | 5 | scutellata |
| MF | N072 | 12 | monticola |
| MF | N072 | 1 | monticola |
| MF | N072 | 2 | monticola |
| MF | N072 | 3 | monticola |
| MF | N072 | 11 | monticola |
| MF | N072 | 14 | monticola |
| MF | N072 | 15 | monticola |
| MF | N073 | 4 | monticola |
| MF | N078 | 13 | monticola |
| MF | N078 | 1 | monticola |
| MF | N078 | 2 | monticola |
| MF | N078 | 3 | monticola |
| MF | N078 | 11 | monticola |
| MF | N078 | 12 | monticola |
| MF | N078 | 14 | monticola |
| MF | N078 | 15 | monticola |
| MF | N081 | 13 | monticola |
| MF | N081 | 14 | monticola |
| MF | N081 | 15 | monticola |
| MF | N084 | 1 | monticola |
| MF | N084 | 5 | monticola |
| MF | N085 | 13 | monticola |
| MF | N085 | 14 | monticola |
| MF | N085 | 15 | monticola |
| MF | N087 | 1 | monticola |
| MF | N087 | 5 | monticola |
| MF | N089 | 13 | monticola |
| MF | N089 | 15 | monticola |
| MF | N089 | 1 | monticola |
| MF | N089 | 2 | monticola |
| MF | N089 | 3 | monticola |
| MF | N089 | 4 | monticola |
| MF | N090 | 1 | monticola |
| MF | N090 | 5 | monticola |
| MKF | N004 | 1 | monticola |
| MKF | N004 | 5 | monticola |
| MKF | N007 | 1 | monticola |
| MKF | N011 | 1 | monticola |
| MKF | N013 | 1 | monticola |
| MKF | N108 | 12 | monticola |
| MKF | N109 | 13 | monticola |
| MKF | N109 | 14 | monticola |
| MKF | N109 | 15 | monticola |
| MKF | N111 | 3 | monticola |
| MKF | N111 | 12 | monticola |
| MKF | N111 | 13 | monticola |
| MKF | N111 | 14 | monticola |
| MKF | N111 | 15 | monticola |
| MKF | N112 | 2 | monticola |
| MKF | N112 | 13 | monticola |
| MKF | N112 | 14 | monticola |
| MKF | N112 | 15 | monticola |
| MKF | N113 | 1 | monticola |
| MKF | N113 | 12 | monticola |
| MKF | N113 | 15 | monticola |
| MKS | N055 | 12 | scutellata |
| MKS | N055 | 13 | scutellata |
| MKS | N055 | 14 | scutellata |
| MKS | N055 | 10 | scutellata |
| MKS | N056 | 13 | scutellata |
| MKS | N056 | 15 | scutellata |
| MKS | N056 | 1 | scutellata |
| MKS | N060 | 11 | scutellata |
| MKS | N060 | 12 | scutellata |
| MKS | N060 | 14 | scutellata |
| MKS | N060 | 15 | scutellata |
| MKS | N069 | 13 | scutellata |
| MKS | N069 | 14 | scutellata |
| MKS | N069 | 15 | scutellata |
| MKS | N069 | 11 | scutellata |
| MKS | N070 | 11 | scutellata |
| MKS | N070 | 12 | scutellata |
| MKS | N070 | 13 | scutellata |
| MKS | N070 | 14 | scutellata |
| MKS | N070 | 15 | scutellata |
| MS | N091 | 12 | scutellata |
| MS | N091 | 13 | scutellata |
| MS | N091 | 14 | scutellata |
| MS | N091 | 15 | scutellata |
| MS | N091 | 11 | scutellata |
| MS | N092 | 12 | scutellata |
| MS | N092 | 13 | scutellata |
| MS | N092 | 14 | scutellata |
| MS | N092 | 15 | scutellata |
| MS | N101 | 13 | scutellata |
| MS | N101 | 14 | scutellata |
| MS | N101 | 15 | scutellata |
| MS | N101 | 12 | scutellata |
| MS | N103 | 14 | scutellata |
| MS | N103 | 15 | scutellata |
| NHF | N017 | 12 | monticola |
| NHF | N017 | 11 | monticola |
| NHF | N017 | 13 | monticola |
| NHF | N017 | 14 | monticola |
| NHF | N017 | 15 | monticola |
| NHF | N018 | 13 | monticola |
| NHF | N018 | 12 | monticola |
| NHF | N018 | 14 | monticola |
| NHF | N018 | 15 | monticola |
| NHF | N022 | 1 | monticola |
| NHF | N022 | 12 | monticola |
| NHF | N022 | 13 | monticola |
| NHF | N022 | 14 | monticola |
| NHF | N022 | 15 | monticola |
| NHF | N023 | 12 | monticola |
| NHF | N023 | 14 | monticola |
| NHF | N023 | 15 | monticola |
| NHF | N023 | 11 | monticola |
| NHF | N023 | 13 | monticola |
| NHF | N024 | 1 | monticola |
| NHF | N045 | 1 | monticola |
| NHF | N046 | 12 | monticola |
| NHF | N046 | 14 | monticola |
| NHF | N046 | 6 | monticola |
| NHF | N046 | 13 | monticola |
| NHF | N046 | 15 | monticola |
| NHF | N048 | 1 | monticola |
| NHF | N050 | 2 | monticola |
| NHS | N026 | 11 | scutellata |
| NHS | N026 | 13 | scutellata |
| NHS | N026 | 14 | scutellata |
| NHS | N026 | 15 | scutellata |
| NHS | N029 | 11 | scutellata |
| NHS | N029 | 12 | scutellata |
| NHS | N029 | 13 | scutellata |
| NHS | N029 | 14 | scutellata |
| NHS | N029 | 15 | scutellata |
| NHS | N030 | 12 | scutellata |
| NHS | N030 | 13 | scutellata |
| NHS | N033 | 11 | scutellata |
| NHS | N033 | 12 | scutellata |
| NHS | N033 | 13 | scutellata |
| NHS | N033 | 14 | scutellata |
| NHS | N033 | 15 | scutellata |
| NHS | N043 | 12 | scutellata |
| NHS | N043 | 13 | scutellata |
| NHS | N043 | 14 | scutellata |
| NHS | N043 | 15 | scutellata |
| NHS | N043 | 11 | scutellata |
